# Analysis of *R* Genes Related to Blackcurrant Reversion Virus Resistance in the Comparative Transcriptome of *Ribes nigrum* cv. Aldoniai

**DOI:** 10.3390/plants11223137

**Published:** 2022-11-16

**Authors:** Ana Dovilė Juškytė, Ingrida Mažeikienė, Vidmantas Stanys

**Affiliations:** Lithuanian Research Centre for Agriculture and Forestry, Institute of Horticulture, Kaunas Str. 30, 54333 Babtai, Lithuania

**Keywords:** blackcurrant reversion virus, dominant resistance, *R* gene, unigene

## Abstract

Blackcurrant reversion virus (BRV) is the most destructive mite-transmitted pathogen in blackcurrants. The understanding of the resistance to BRV is limited, hindering and delaying the selection process. To identify the resistance (*R*) gene for BRV resistance, a gene expression analysis based on de novo blackcurrant cv. Aldoniai comparative transcriptome analysis (mock- and BRV-inoculated samples at 2 and 4 days post-inoculation (dpi)) was performed. In this study, 111 annotated clusters associated with pathogenesis according to conservative *R* gene domains were identified. In virus-infected samples, only Cluster-12591.33361 showed significant expression at 4 dpi. The expression profiles of this cluster were significantly associated with the presence of BRV particles in plant tissues, making it a putative *R* gene in the dominant resistance strategy in the BRV–*Ribes nigrum* interaction. The newly identified gene *R.nigrum*_*R* belongs to the CC-NBS-LRR class and has 63.9% identity with *RPM1* in *Populus* spp. This study provides new insights on dominant putative *R* genes related to resistance to BRV in *R. nigrum*, which could aid targeted research and genetic improvement in breeding programs of blackcurrants.

## 1. Introduction

Biotechnological and plant genetic research approaches have helped to elucidate the mechanisms of the immune response against viruses in plants. Several evolutionary strategies in response to viral infection have been identified in plants. Resistance induced by dominant and recessive resistance (*R*) genes, as well as hormone-mediated resistance and RNA interference pathways, has been analyzed [1]. Many dominant *R* genes belonging to the nucleotide-binding site leucine-rich repeat family (encoding proteins NBS-LRR) have been identified in plants. Intracellular NBS-LRR proteins are divided into two classes: TIR-NBS-LRR (TNL) proteins containing the Toll/Interleukin-1 (TIR) receptor domain and non-TIR-NBS-LRR (non-TNL) proteins that lack the TIR domain. Members of the non-TNL class usually contain a predicted coiled-coil (CC) domain and belong to the CC-NBS-LRR (CNL) class [2,3]. It is known that TNLs can provide resistance to diseases in solanaceous and brassicaceous species, while CNLs are more common in dicotyledon and cereal crops [4,5]. Studies have revealed insights into defense responses to viruses in maize [6], tomato [7], potato [8], tobacco [9], etc.

Blackcurrant reversion virus (BRV) (*Nepovirus* of group C) is spread through the vector *Cecidophyopsis* spp. and causes blackcurrant reversion disease (BRD) during intracellular interaction with host plants [10,11,12,13]. The combination of pest–pathogen–disease causes substantial losses in yield up to 100% [10,14]. Thus, natural plant resistance may prove to be an effective and environmentally friendly method to control BRV spread. Cultivars with genetically determined resistance are the best option for blackcurrant breeding and plantation use. Resistance to the BRV biological vector and, hypothetically, to BRV is known to be inherited from several *Ribes* spp. through dominant genes [15,16,17]. However, the genetic control of BRV resistance, including specific genes, is not entirely known. An effective in vitro inoculation method [18] and comparative transcriptome analysis of cv. Aldoniai, having BRV resistance inherited from *R. dikuscha*, were performed in 2022 [19]. These studies allowed a transcriptome-wide study to find putative *R* genes in blackcurrants.

This study provides a theoretical foundation for the further screening of putative *R* genes in response to BRV infection. Selected genes were characterized based on annotations of functional domains and motifs, as well as expression levels by quantitative real-time PCR (qRT-PCR). This investigation enriches our knowledge of possible genes related to dominant resistance to BRV and lays the groundwork for future crop improvement in *Ribes* breeding programs.

## 2. Results

### 2.1. Conserved Domain and Motif Analysis of Putative R Genes in R. nigrum

**Figure 1:**
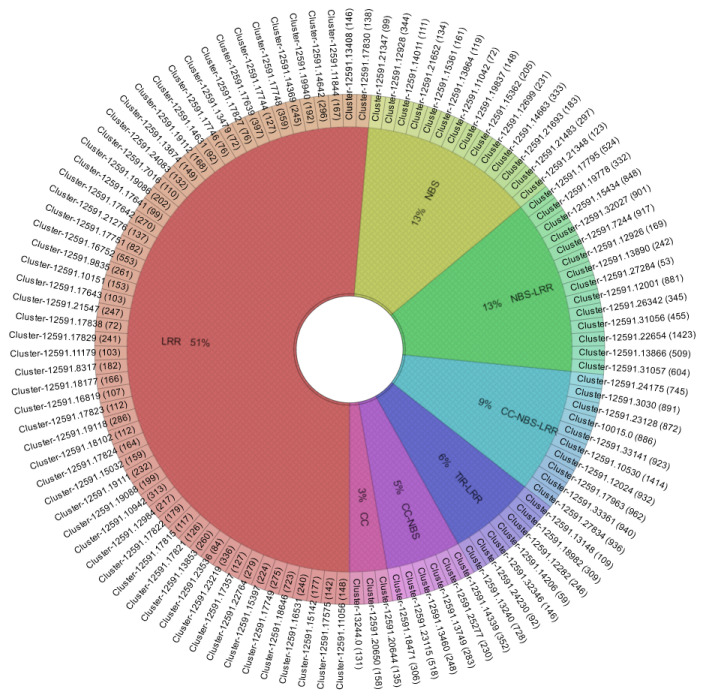
Distribution of predicted conserved domains in 111 unigenes related to BRV pathogenesis in *R. nigrum* cv. Aldoniai. The length of the sequences of amino acids is indicated in brackets, and amino acid sequences of unigenes are provided in Table S1.

The availability of the *R. nigrum* cv. Aldoniai transcriptome made it possible to identify and characterize putative *R* genes associated with resistance to BRV. Initially, all 48,966 identified unigenes in blackcurrants were scanned by selecting sequences related to pathogenesis and disease resistance, which resulted in the identification of 111 candidate proteins (Table S1). The sorting analysis confirmed the presence of 111 unigenes containing at least one conserved domain specific to *R* genes: CC, TIR, NBS, and LRR. Figure 1 shows blackcurrant sequences classified into seven different groups based on conserved domains.

This analysis revealed that 40% of sequences had highly conserved NBS domains. These NBS-encoding unigenes were categorized into four groups according to the presence of conserved domains: NBS (13%), NBS-LRR (13%), CC-NBS (5%), and CC-NBS-LRR (9%). Moreover, more than half of unigenes had partial sequences (a problem due to de novo transcriptome assembly without a reference genome) and encoded only one domain from the 5′ or 3′ end of the resistance gene: LRR (51%) and CC (3%). In addition, 6% of unigenes that lacked the NBS domain were found to contain both TIR and LRR domains. Generally, *R* genes are characterized by the presence of three domains in their sequences: a variable amino-terminal domain, a central NBS domain, and a carboxy-terminal LRR domain. Thus, from the identified *R* gene candidates in *R. nigrum*, the complete gene structure was identified in 10 sequences belonging to the CC-NBS-LRR class.

**Figure 2:**
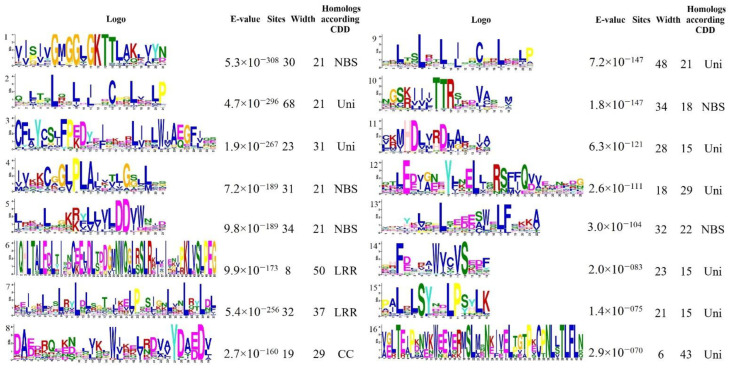
Sequence logos of 16 conserved motifs identified in 111 putative *R* genes of *R. nigrum*. Sequence logo representation was generated from multiple alignments with MEME software. CDD—Conserved Domain Database; NBS—nucleotide-binding site; LRR—leucine-rich repeat; CC—coiled-coil domain; Uni—unidentified motif, unique to *R. nigrum* cv. Aldoniai.

Furthermore, the conserved motifs present in the selected 111 protein sequences were analyzed using the MEME suite (Figure 2 and Figure S1). A total of 16 conserved motifs were identified, each consisting of ≥ 15 amino acids. According to the Conserved Domain Database (CDD), eight motifs were identical to conserved domains: five motifs (Nos. 1, 4, 5, 10, and 13) were specific to the NBS domain, motifs No. 6 and 7 were identical to the LRR domain, and No. 8 was identical to the CC domain. Interestingly, in addition to known conserved motifs, our analysis identified eight motifs that had not been identified according to the CDD database, so they are unique to blackcurrants. *Ribes* spp. are systemically distinct from other plant species, which may have led to mutations in motifs compared to other plants. For example, unigenes containing LRR domains had unidentified (in statistical analysis) leucine-rich repeat Nos. 2, 9, and 16, while unigenes with the NBS domain had motif Nos. 12, 14, and 15 (Figure 1 and Figure 2). Thus, these motifs are likely to be specific to the LRR or NBS domains, respectively, in blackcurrants.

### 2.2. Expression Patterns of Unigenes in Response to BRV Infection

**Figure 3:**
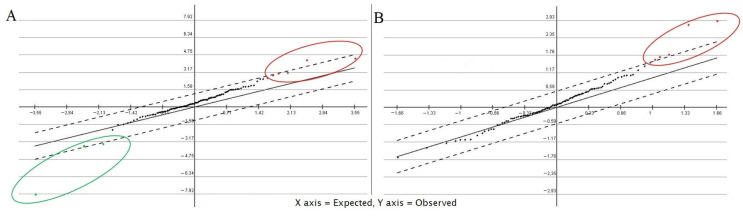
Statistical analysis of 111 unigenes according to FPKM values related to BRV resistance in *R. nigrum* cv. Aldoniai at 2 dpi (**A**) and 4 dpi (**B**). The SAM graphs were generated by MeV v. 4.8 package. Red dots indicate up-regulated genes, and green dot indicate down-regulated genes.

The selected unigenes were differentially expressed during the entire period of the experiment. The statistical analysis of the FPKM values of 111 clusters reduced the number to 14 statistically significant unigenes in SAM graphs—8 unigenes at 2 dpi and 6 at 4 dpi (Figure 3A,B).

**Figure 4:**
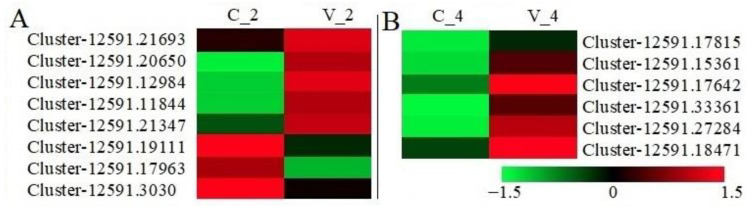
Heatmaps of 8 significantly expressed genes at 2 dpi (**A**) and 6 significantly expressed genes at 4 dpi (**B**). C_2 and C_4—the average of three biological replicates of mock-inoculated plants at 2 and 4 dpi, respectively. V_2 and V_4—the average of three biological replicates of BRV-inoculated plants at 2 and 4 dpi, respectively. In the scale bar, green color indicates low (−1.5) gene expression values, and red color indicates high expression (1.5).

To visualize the differentially expressed genes in response to a virus attack, a selected set of genes are presented in heatmaps. Figure 4 represents only the 14 significant expression patterns according to FPKM values among mock-inoculated and BRV-inoculated plants at 2 and 4 dpi (Figure 4). On the second day after BRV infection, five unigenes (Cluster-12591.21693, Cluster-12591.20650, Cluster-12591.12984, Cluster-12591.11844, and Cluster-12591.21347) were significantly up-regulated, while three unigenes (Cluster-12591.19111, Cluster-12591.17963, and Cluster-12591.3030) were down-regulated. At 4 dpi, the number of reliably expressed genes decreased to six (Cluster-12591.17815, Cluster-12591.15361, Cluster-12591.17642, Cluster-12591.33361, Cluster-12591.27284, and Cluster-12591.18471), all of which showed up-regulated expression patterns. Only three unigenes with the structure CC-NBS-LRR (Cluster-12591.17963 and Cluster-12591.3030 were down-regulated at 2 dpi, and Cluster-12591.33361 was up-regulated at 4 dpi) were found. Other unigenes were partial genes comprising one or two conserved domains.

The origins of the 14 significantly expressed genes were identified using the NCBI Blast database (Table 1). The majority of them showed the highest identity with disease resistance protein family *RGAs*. In addition, representatives from *At1g12280*, *RPM1*, *RPP13*, and *TVM resistance protein* families were found in blackcurrants. The highest matching percent (70%) was detected between Cluster-12591.27284 and *TMV resistance protein N* in *Pyrus* x *bretschneideri*.

**Figure 5:**
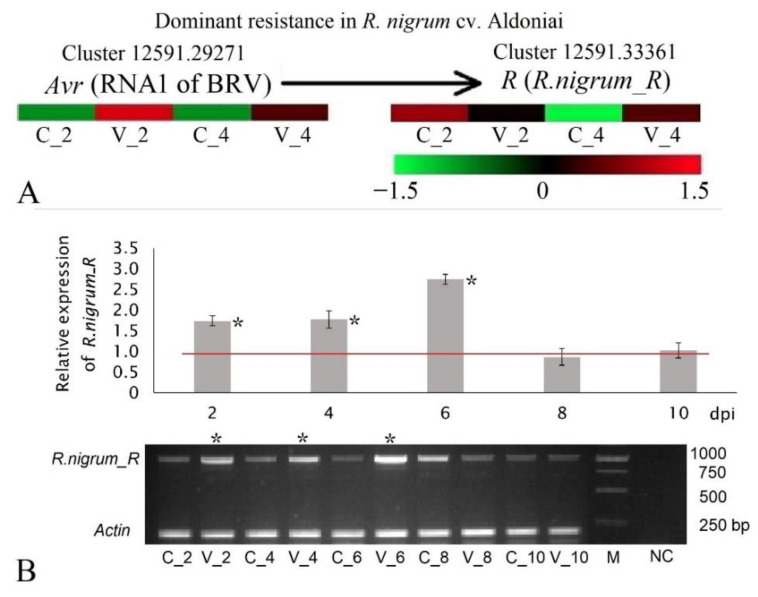
Gene expression in *Avr*-*R* resistance system of blackcurrant cv. Aldoniai based on RNA-seq (**A**) and qRT-PCR (**B**). Control (mock-inoculated at 2–10 dpi) samples C_2–C_10; treated (BRV-inoculated at 2–10 dpi) samples V_2–V_10; M—mass ruler, NC—negative control in the agarose gel, *—treatment with positive qRT-PCR reaction of BRV. Significant expression level log2FC > 1 in heatmap (**A**); significant expression level 2^−ΔΔC^_T_ > 1 is separated by a red line in the bar chart (**B**).

In accordance with the FPKM value transformed to log2FC during the entire period of the experiment (2 and 4 dpi), genes were divided into two groups: those having an increasing expression pattern (Cluster-12591.19111, Cluster-12591.17963, Cluster-12591.3030, Cluster-12591.17815, Cluster-12591.15361, Cluster-12591.17642, Cluster-12591.33361, Cluster-12591.27284, and Cluster-12591.18471) and those having a decreasing expression pattern (Cluster-12591.21693, Cluster-12591.20650, Cluster-12591.12984, Cluster-12591.11844, and Cluster-12591.21347). Of all of these genes, only Cluster-12591.33361 with the structure CC-NBS-LRR plus six motifs unique to *R. nigrum* (Figure S1) showed significant expression at 4 dpi (log2FC varied from −0.40 to 1.09), making it a potential candidate for an *R* gene in blackcurrant cv. Aldoniai (Table 1 and Figure 5A).

### 2.3. Expression and Phylogenetic Analyses of Putative R Gene to BRV Resistance

Based on the previous analysis, Cluster-12591.33361 was identified as a representative of *Resistance to Pseudomonas syringae* pv. *maculicola 1* (*RPM1*) and was selected as the potential resistance candidate to BRV in *R. nigrum* cv. Aldoniai (Figure 5). According to transcriptome analysis data, the expression levels of Cluster 12591.33361 were detected at 2 and 4 dpi (log2FC −0.40 and 1.09, respectively). The avirulence (*Avr*) factor for *RPM1* is RNA1 of BRV (log2FC 12.85 and 11.29 at 2 and 4 dpi, respectively) (Figure 5A). To elucidate the presence and function of this gene in later periods (2–10 dpi) of BRV infection, an expression profile analysis via qRT-PCR was performed in BRV-resistant cv. Aldoniai and BRV-susceptible cv. Ben Tirran. In the BRV-susceptible genotype, the virus was detected during the entire period of the experiment, and the expression levels of *R.nigrum_R* compared to the control were not determined (data not presented). Meanwhile, in the resistant genotype, viral infection was detected until 6 dpi (Figure 5B). The relative expression levels of *R.nigrum_R* directly correlated with the presence of BRV in microshoots and were significantly higher at 2, 4, and 6 dpi in comparison to mock-inoculated plants of *R. nigrum* cv. Aldoniai. When BRV was no longer detected in microshoots (8 and 10 dpi), the expression levels of *R.nigrum_R* decreased. The expression levels of putative *R.nigrum_R* in different RNA-seq and qRT-PCR experiments were not consistent in cv. Aldoniai at the beginning of the infection (at 2 dpi). This slight difference might be due to errors in different inoculation experiments.

**Figure 6:**
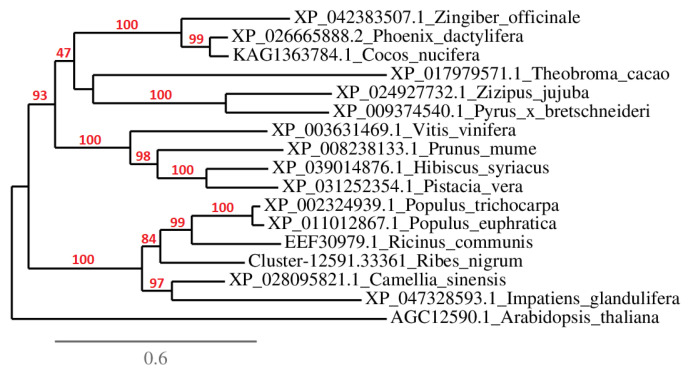
Phylogenetic tree of 17 *RPM1* homologs at the amino acid level in different plant species, performed using the PhyML program. The labels of members in the phylogenetic tree consist of accession numbers in NCBI database_plant species.

The phylogenetic relationships and genetic polymorphism of the *RPM1* family were inferred by constructing a phylogenetic tree using full-length amino acid sequences of *RPM1* homologs from different plant species together with Cluster-12591.33361 identified in the transcriptome of cv. Aldoniai (Figure 6). Two reliably distinct branches at 93% and 100% bootstraps separated it from the *Arabidopsis thaliana RPM1* homolog. The sequence detected in *R. nigrum* was grouped into the phylogenetic dendrogram’s second branch with *RPM1* homologs from *Populus* spp., *Ricinus communis, Citrus sinensis, Camellia sinensis,* and *Impatiens glandulifera*. In this branch, *RPM1* homologs were genetically diverse and reliably separated into several sub-branches, which shows their uniqueness. The *RPM1* homolog of *R. nigrum,* showing a clear correlation with BRV resistance, was in a separate phylogenetic branch at 84% bootstrap and showed high genetic diversity from resistance genes to different pathogens in other plants.

## 3. Discussion

The pathogenic complex of the virus (BRV), its biological vector (the gall mite), and blackcurrant reversion disease cause a significant or complete reduction in berries’ yield and quality. Therefore, a prime goal of blackcurrant breeders is to develop BRV-resistant cultivars. To achieve this goal, it is necessary to identify genes that determine resistance to this harmful pathogen. Numerous genome-wide investigations of the NBS-encoding gene family have been performed in plants, including Arabidopsis [5], blueberry [20], apple [21], wheat [22], cabbage [23], etc., and have demonstrated their importance in searching for specific *R* genes to various pathogens.

Dominant resistance through the *R* gene plays an important role in plant defense against viral pathogens during the first infection period [1]. However, to date, no research has been conducted on such a gene family in blackcurrant, an important horticultural plant that is grown in temperate climate zones of Europe, Asia, New Zealand, Australia, and North America. In this study, a transcriptome-wide search uncovered 111 unigenes related to pathogenesis in *R. nigrum*, which provides a useful database of potential resistance genes for further testing in the development of marker-assisted strategies for the selection of BRV resistance (Figure 1). For example, 16 resistance genes of the TIR-NBS-LRR class were found to be involved in resistance to turnip mosaic virus in cabbage [24]. The NBS domain is responsible for the binding and hydrolysis of ATPases during plant disease resistance. Meanwhile, the LRR domain is involved in protein–protein interactions and is important in the recognition of pathogen-derived avirulence factors [25]. Multiple studies of molecular and functional analyses of plant genetics have shown that *R* genes may contain sequences related to functions at the amino acid level but not identical at the nucleic acid level [26]. In this study, we identified not only the conserved domains CC, TIR, NBS, and LRR, characteristic of *R* genes, in blackcurrant unigenes (Figure 1) but also unique functional sequences. Eight unique motifs in amino acids among resistant unigenes in *R. nigrum* were identified (Figure 2 and Figure S1), and several of them are putative NBS or LRR domains. An explanation for this is that the evolutionarily conserved LRR domain is associated with innate immunity in many proteins, not only in Plantae but also in Animalia [27].

Fourteen candidate unigenes with significant expression were identified as responding to BRV infection (Figure 3). Three unigenes (Cluster-12591.17963, Cluster-12591.3030, and Cluster-12591.33361) with complete *R* gene structures showed expression in the de novo assembled transcriptome of BRV-resistant cv. Aldoniai (Figure 4). According to expression data, only Cluster-12591.33361 had up-regulated expression at 4 dpi and was used for deeper analysis as a putative *R* gene for BRV, referred to as *R.nigrum_R*. The sequence of this gene at the amino acid level (Table S1) was identified as a homolog to the disease resistance protein *RPM1* in *R. nigrum* and had 63.88% identity with *RPM1* in *Populus trichocarpa* [28] (Figure 6). Based on the structural features, *RPM1* encodes a CC domain at the N-terminus, a central NBS domain, and a C-terminal LRR [29] and is a typical member of the CC-NBS-LRR class; our studies also confirmed this fact (Figure 1). However, putative molecular motifs/functional LRR domains unique in the *R.nigrum_R* gene have also been identified and result in the genetic diversity of blackcurrants in comparison to other plants.

Dual or multiple specificities of *R* genes can be explained by the guard hypothesis [30] that an *R* gene guards a single avirulence factor that is modified by multiple effectors. One of the examples is *RPM1*, which recognizes different effectors of the same bacteria [31]. In Arabidopsis, *RPM1* confers resistance against the bacterium *Pseudomonas syringae* pv. *maculicola* expressing *AvrRpm1* or *AvrB*. Pathogen avirulence effectors induce *RIN4* (*RPM1-interacting protein 4*) phosphorylation, which is required for the activation of the *RPM1*-induced defense response, leading to a rapid hypersensitive response (HR) and the restriction of pathogen growth [32]. The increasing expression patterns of *RPM1* have been studied not only in response to bacteria [33] but also to fungi [34] and viruses [35]. In this study, qRT-PCR was used to establish the expression profiles of *RPM1* during BRV infection in blackcurrants. We determined that the expression of *RPM1* directly correlated with the presence of the virus in BRV-resistant plants (Figure 5A). BRV can be monitored by PCR in inoculated microshoots of cv. Aldoniai up to the 6–8 dpi period. The virus can persist in individual microshoots of this BRV-resistant cultivar for up to 8 dpi, but in many cases, it lasts for up to 6 dpi using the inoculation method under in vitro conditions [18]. In this study, BRV infection was detected in pooled samples until 6 dpi (Figure 5B). Dominant resistance determined by the *RPM1* homolog in blackcurrants (Cluster-12591.33361), referred to as *R.nigrum_R*, relies on the interaction between the avirulence factor RNA1 of BRV (Cluster-12591.29271) [19] and a specific *R* gene product CC-NBS-LRR, which specifically recognizes the *Avr* factor in the *Avr*-*R* system (Figure 5A). This dominant resistance mechanism was effective six days after the virus’s mechanical transmission into the blackcurrant microshoots. In parallel, phytohormone-mediated resistance induced by salicylic acid, jasmonic acid, auxin, and ethylene synthesis in cv. Aldoniai (a virus- and gall-mite-resistant interspecific hybrid) was also observed due to BRV infection [19].

## 4. Materials and Methods

### 4.1. Prediction of Putative R Genes in Transcriptome of R. nigrum cv. Aldoniai

RNA transcripts of *R. nigrum* cv. Aldoniai were used for the prediction of *R* gene candidates for BRV resistance in blackcurrants (raw data are available on BioProject PRJNA797914 in the NCBI database). Comparative analysis of the de novo assembled transcriptome of mock-inoculated (control) and BRV-inoculated (treatment) microshoots at 2 and 4 days post-inoculation (dpi) was performed [19]. From 48,966 functionally annotated unigenes identified by NCBI Blast and the Conserved Domain Database (CDD) [36], specific domains (TIR, CC, NBS, and LRR) associated with pathogenesis-related or disease-resistant genes were detected in 113 unigenes. This set of sequences was further filtered by applying a cut-off of the FPKM (fragments per kilobase of transcript per million mapped reads) expression value of 1.0. Sequences with lower expression values were discarded; 111 sequences were left.

The distribution of putative *R* genes according to the conserved domain was determined using Krona2 [37]. The structural diversity of specific motifs in unigenes was performed using Multiple Expectation Maximization for Motif Elicitation (MEME) suite 5.4.1, choosing 16 motifs to find with an E-value < 1 × 10^−10^ [38]. A SAM (significant analysis for microarrays) graph was constructed according to two-class unpaired FPKM data of unigenes using value parameters by the method in [39], the K-nearest neighbors (10) imputer with Multiple Experiment Viewer (MeV v 4.8) package [40], and an adjusted *p*-value of < 0.05. Heatmaps (HMs) of the unigenes were constructed using transcriptome data using the R packet; the FPKM values were transformed to log2FoldChange (log2FC). Changes in expression levels were also evaluated by log2FC values; log2FC ≥ 1 was considered to show significant expression.

### 4.2. Expression and Phylogenetic Analyses of R.nigrum_R

In vitro–propagated microshoots of *R. nigrum* cvs. Aldoniai and Ben Tirran were used in the inoculation assay and the expression of *R.nigrum_R*. Inoculum preparation, plant material preparation, and the inoculation assay with BRV through roots were performed identically to the methods described by Juškytė et al. [18]. Inoculated plants were cultivated for 2, 4, 6, 8, and 10 dpi. Mock- and BRV-inoculated microshoots in 3 replicates were stored in liquid nitrogen until RNA extraction. Total RNAs were extracted using the GeneJET Plant RNA Purification Mini Kit according to the manufacturer’s protocol (Thermo Scientific, Vilnius, Lithuania). cDNAs were synthesized with the Maxima H Minus First Strand cDNA Synthesis Kit using oligo d (T)_20_ primers and 50 ng of RNA according to the manufacturer’s protocol (Thermo Scientific, Vilnius, Lithuania). cDNAs were stored at −20 °C until the qRT-PCR.

The primers *R.nigrum*_*R_*F 5′atcaccttaccgaatgcatgttt3′ and *R.nigrum*_*R_*R 5′ctcgagaagataaagcagctcag3′, *Actin*_F 5′tcaactatgttccctggtattgc3′, and *Actin*_R 5′ctcccttggaaatccacatctg’3 were designed with the Primer3Plus program, version 3.2.6 (Cambridge, MA, USA) [41]. Primers suggested by Lemmetty et al. [42] were used for BRV detection. The qRT-PCR reaction was performed in a 20 µL reaction volume containing 10 μL of MyTaq Mix 2x (Bioline GmbH, Luckenwalde, Germany), 2 μL of 20x EvaGreen dye (Biotium, Inc., Fremont, CA, USA), 1 µL of cDNA, and 10 pmol of each forward and reverse primer. The analysis was carried out on three biological replicates on a CFX 96 Deep Well Real-Time System (Bio-Rad, Hercules, CA, USA) under the following conditions: 95 °C for 3 min; 40 cycles of 95 °C for 20 s, 58 °C for 20 s, and 72 °C for 30 s; 72 °C for 5 min; and step 95 °C–65 °C for 20 min was inserted for identification of melting curve.

Relative expression was assessed by the 2^−ΔΔC^_T_ method [43] using *Actin* as the internal control gene. Means and SEM (standard error of the mean) from independent experiments were subjected to STAT-ENG. The visualization of specific fragments (992 bp of *R.nigrum*_*R*, 175 bp of *Actin*, and 481 bp for BRV detection (marked as an asterisk in Figure 5B)) was performed in 1.2% agarose gel using ethidium bromide staining and UV illumination.

Phylogenetic analysis of the *R. nigrum RPM1* homolog and 16 homologous sequences in other plants (NCBI database) was performed using the maximum likelihood method implemented in the PhyML program; each branch was assessed by bootstrap analysis with 100 replicates [44].

## 5. Conclusions

The present study provides comprehensive insights into 111 transcriptome-wide virus-responsive unigenes in *R. nigrum*. These genes, especially Cluster-12591.33361, referred to as *R.nigrum_R*, and their verification should be the focus of subsequent work on resistance genetics, thereby providing opportunities for improving BRV resistance. In addition, they could be used as candidates for engineering BRV resistance in *R. nigrum* and creating BRV-resistant cultivars.

## Figures and Tables

**Table 1:** Identification and log2FC expression values of significantly expressed *R. nigrum* genes.

Cluster	Accession No. and Gene Name According to NCBI Blast	Gene Identity, %	log2FC (V_2vsC_2)	log2FC (V_4vsC_4)
Cluster-12591.21693	XP_028108391_*RGA4* [*Camellia sinensis*]	54.01	0.41	−0.48
Cluster-12591.20650	KAB1227433_*RGA4* [*Morella rubra*]	54.01	0.93	−0.22
Cluster-12591.12984	XP_021660273_*RGA1* [*Hevea brasiliensis*]	52.83	0.50	0.01
Cluster-12591.11844	XP_002526758_*RGA4* [*Ricinus communis*]	60.00	0.77	0.30
Cluster-12591.21347	XP_022735032_*At1g12280* [*Durio zibethinus*]	63.33	0.60	−0.79
Cluster-12591.19111	XP_034689147.1_*RGA3* [*Vitis riparia*]	52.32	−0.80	−0.40
Cluster-12591.17963	XP_030942204.1_*RPM1* [*Quercus lobata*]	51.28	−0.45	−0.42
Cluster-12591.3030	XP_002281054.1_*RPP13* [*Vitis vinifera*]	50.63	−0.71	0.03
Cluster-12591.17815	XP_023923535.1_*TMV resistance protein N* [*Quercus suber*]	54.31	0.06	0.40
Cluster-12591.15361	KAB1200960.1_*RGA3* [*Morella rubra*]	52.03	−0.72	0.81
Cluster-12591.17642	XP_015865709.2_*RGA1* [*Ziziphus jujuba*]	34.23	0.24	0.50
Cluster-12591.33361	XP_002324939.1_*RPM1* [*Populus trichocarpa*]	63.88	−0.40	1.09
Cluster-12591.27284	XP_009335301.1_*TMV resistance protein N* [*Pyrus* x *bretschneideri*]	70.00	0.07	0.43
Cluster-12591.18471	XP_021663085.1_*RGA3* [*Hevea brasiliensis*]	58.05	0.04	0.49

## Data Availability

The study did not report any data.

## Supplementary Materials

The following supporting information can be downloaded at: https://www.mdpi.com/article/10.3390/plants11223137/s1. Figure S1: Conserved motifs in 111 unigenes of R. nigrum cv. Aldoniai; Table S1: Amino acid sequences of putative *R* genes in transcriptome of *R. nigrum* cv. Aldoniai.
