# Supplementary material for: Analysis of R Genes Related to Blackcurrant Reversion Virus Resistance in the Comparative Transcriptome of Ribes nigrum cv. Aldoniai
Source: Plants (Basel). 2022 Nov 16;11(22):3137. doi: 10.3390/plants11223137 (PMC9692259; doi:10.3390/plants11223137)
Supplement: Supplementary file 1 [file plants-11-03137-s001.zip › Table S1.pdf]

Table S1. Amino acid sequences of putative *R* genes in transcriptome of *R. nigrum* cv. Aldoniai

1.	**>Cluster-12591.12926;orf1 len=169 frame:-2 start:509 end:3 1173804735|XP_020532758.1 probable disease resistance protein At1g12280 [Jatropha curcas]**<br>IKEEVDRAAKLFAKLKEEKRYVLIFDDVWEAFNLADVGIPEPTEGNGCKLILTTRSLEVCRRMECKDIKMELFSEDEAWNLFLEKVGRDVIMTSGLENIAKQVAEECACLPLAIVTVGTSIKGVVDEHEWRTALRKLRECTKGFEGMSKVIEQLKYSYSQLNNEPAQRC
2.	**>Cluster-12591.11056;orf1 len=148 frame:2 start:2 end:445 1105500079|XP_019077551.1 PREDICTED: probable disease resistance protein At1g12280 [Vitis vinifera]**<br>KGKFFVQAGVELTEIPKNVKWEEVERMSLMQNKIVELTGTPKCTNLSTLFLNDNKELEKITGAFFQSMPILRVLDLSRNCINELPKEICALVSLQYLNLSSMCLEELPVEFKNLVNLKYLNLNLSGIRIIPEGIIANLKNLQVLYILG
3.	**>Cluster-12591.12928;orf1 len=344 frame:-3 start:1034 end:3 802555275|XP_012065344.1 probable disease resistance protein At1g12280 [Jatropha curcas]**<br>SLIATYYCATTSIKPRDIVVMDIFSPLIEIGKMIYTPISKCVKYHRGLDESMKILQEKLEDLTVRKTDIESQIRAERRSDKNPKMEVDRWLVKVQKINDDKELEKEVAKGKFLSRSRLGRRAAAKIREVEVLHQEGSFSDGLLVDAIPSRGEMFPATTLVGKATSKKVEEIWNSLFSVNVLKIGVWGMGGSGKTTIMKLIHNRVLKQTDRFDNVYWITVSKETSVFKLQDGMASALKLEGFKKIKEEVDRAAKLFAKLKEEKRYVLIFDDVWEAFNLEDIGIPEPTIGNGCKLILTTRSLDVCRRIGCKEIKMELLSEDEAWNLFLEKVGRDVIMTSGLEKIAK
4.	**>Cluster-12591.17575;orf1 len=142 frame:2 start:2 end:427 359482672|XP_003632805.1 PREDICTED: probable disease resistance protein At1g12280 [Vitis vinifera]**<br>KGKFFVQAGVELTEIPKNVKWEEVERMSLMENKIVELTGTPECPNLLTLFLNKNDNLEIIDAFFQSMPTLRVLDLSMNSIRRLPKEICELVSLQYLNLSDTNLEELPIELKNLVNLKYLNLLYLGTCIIPEGVVANLIKLQE
5.	**>Cluster-12591.21347;orf1 len=99 frame:2 start:2 end:298 1269931408|XP_022714852.1 probable disease resistance protein At1g12280 [Durio zibethinus]**<br>NKFILFLDDIWDRFDLTKTGIPLPNQSNKSKVVFTTRSKDVCGWMRANKKVEMKCLTEEDALKLFKENVGDDTLNAHPKIHELAKVVAKECVWRDGSL*
6.	**>Cluster-12591.15142;orf1 len=177 frame:-1 start:532 end:2 1105500079|XP_019077551.1 PREDICTED: probable disease resistance protein At1g12280 [Vitis vinifera]**<br>VELTEIPMNVKWEEVERMSLMKNEIVELTGTPKCPNLLTLFLNDNKYLGKITDAFFQSMPTLLVLDLSMNNNIRRLPKEIWELVSLQYLNLSYTNLKELPIELKNLVNLKYLNLHSWGTYIIPEGVVANLIKLQVLYMVGCDVGETLVKELESLKYLNRLSITINTASILSYFLCSP
7.	**>Cluster-12591.17795;orf1 len=524 frame:-3 start:1574 end:3 1344062832|XP_023915001.1 putative disease resistance protein At1g50180 [Quercus suber]**<br>CVSQQFQERDIWEAILIKLVAPSDSQREKFSKMRPDEIAKELFQFQQQKKCLVILDDIWSVEAWDTLKPAFPTGNASNSKLVLTTRIKTIALHVNPRGILEPQCLTEEESWELLQKKAFTRNDYKDLKINREMEELGRKMVERCAGLPLAINVLGGLLQTKDSIDEWRSVHENIDLYLRRGNRHEQGLSRVHGILALSYHELPYFLKPCFLYLSHFQEDEEIRTKNLFRLWVAEGFISEVETKGMEEDVAESYMHELINRSMVQVGTIVKSGRVKTCRIHDLMRDLCLSEANQESFFKNVVYDGNGKVVSPSVRRLAVYSAYQHVKYPRDFELTPKLKSFFLISNKLDYGEGGPLISRINCFKMLRTLTFTRCRFKDGKCKEIGFLIHLRYLSIIECGLRSLPSSIENLRYLQTLFINEYSITVPNEIGKLEQLRHLVLQTSFLEKKCQKLVRLDNLRNLQTLRISSYGDVFDTNGLVRFTNLKNLYIRGWLSKLPEHQHFPPNLTKLTLKSTHSKEDPMATLE

8.	**>Cluster-12591.14339;orf1 len=352 frame:1 start:64 end:1119 1343992178|XP_023878106.1 putative disease resistance protein At1g50180 [Quercus suber]**<br>KKKKMVDAVASFVVERVGDLLTQEAISLGRVRGQVEEMQKELKRMICFLKDADKRQDEDERVKGWISEIREVAYDVEDVIETFALGVTSRSQPGFR<br>NKCKRYASIFIEGARLHKVGSDIADLNSRITNLTGSLERYGIRIKDEGEASSSSVSRRQQKLRRTYSHVVEEYTVGLEKNTENLVRQLVAEEKKGE<br>VVSICGMGGLGKTTLAKKVYHHDGVRRHFDGFAWACISQQFQPRDIWEGILIKLVAPSERQRIQNMRREEIAKELYQFQQQKKCLVILDDIWSVEA<br>WDTLKPAFPTGNASNTKIVLTTRIKTVALHVNPRRFFEPQCLTEEESWKLLQKKAFPRNDYNR*
9.	**>Cluster-12591.14011;orf1 len=111 frame:3 start:3 end:335 1269896381|XP_022759151.1 putative disease resistance protein At1g50180 [Durio zibethinus]**<br>RNMGHEELAKELYQFQQQNKCLVILDDIWSVEAWDTLKPAFPTGNASNGKIVLTTRIKKVASHVNPRGILEPQCLTEEESWELLQKKAFPRNNCTG<br>NLLPNPEVENIMKI*
10.	**>Cluster-12591.25277;orf1 len=230 frame:-3 start:691 end:2 1343992178|XP_023878106.1 putative disease resistance protein At1g50180 [Quercus suber]**<br>KNSFTKKKKKSSRKKKKMAEAVVSFVVERVGDLLIQEVKFLRGVRGQVEQMQTELKRMQRFLKDADKKQGEDELVKGWISEIRELAYDAEDVIEIF<br>ALEVASRSQPGFKNMCKRFATIFTKGAKLHKVGTEIETIKTRIADLTESLQKYGIKFKEESEASSSSVIKRQQIFRRTYSHLVEEYTVGLERNTEI<br>LVSQLVTEEKQGQVISICGMGGLGKTTLANKAYHHDKV
11.	**>Cluster-12591.21652;orf1 len=134 frame:3 start:519 end:920 1269896381|XP_022759151.1 putative disease resistance protein At1g50180 [Durio zibethinus]**<br>RTRLGDIVPSWGENFISLALRFEEMNKRIVDITTGLQTYGIRFNQEGEASTSSVRQRELRRTYSHEIEGDVMGIDKNIEFLVRQLVQGDEPIVSIC<br>GLGGLGKTTLAKQIHSHNEVRRHFDYFAWVCFSKISN*
12.	**>Cluster-12591.15361;orf1 len=161 frame:2 start:374 end:856 1344081147|XP_023924572.1 putative disease resistance protein At3g14460 [Quercus suber]**<br>CNEMIMRSVRTHHLKQLLNKDCWLLFAKHAFDNNNSDKCLELEIIGRKIVEKCTGLPLAAKTLGALLHSKADAVDWYKILESELWELPNDKNDILP<br>ALLLSYHYLPSHLSVVLLTVQFSPRIMSLKRRRLSYCGWPKIFYKILQVQLNKWRKSDMSTSMN*
13.	**>Cluster-12591.22654;orf1 len=1423 frame:2 start:980 end:5248 1162571374|XP_020424109.1 putative disease resistance protein At4g19050 [Prunus persica]**<br>GTLEKSQIFWKEKKKLFLCVHPPLLSGMDPITVKQREEKILALLDNKDVTTIVLVGIPGIGKTWMARKICDQVRKVFDITLWVYLNRKYDKWALYE<br>NIACQLSLLSTTEEKDGDDSDEEVEEHKEDKEEVSLEKLKLKISATLANKRCLLVLDDEGNRMKEEEIMSECKTLLPLNHQNECKVLITTISRDSS<br>HVIEGTKTRIEIVEPLTEEESLQLLQERAEISDAEFSRCEPHLRAVIRKSACLPSAIIMIAEVLKHLRLRKHDVGVWTFERALAEVACNENYHTID<br>LLRSGFDLLPETDMINCCWHSRQFFHDRGSIHYNELIAYWILEGYLGDIDHIEKAYEKGHSILMELVNRGMLTKQEDNFVGVEGALLEVDDHRRDG<br>FNGTSSLGLENVSADGEWGGLGKITWVDGMIKSLSNGGKWQKLSTLLIDGNRLCREVPDLFSQFLGLQVLALFNPRFKSVPLSLSKLENLLVLVLR<br>GCCLLDNVDHLKELNKLTVLEISGASSLTKFPNDLFEKIPNLERLNLSGSQVKELPSSIENLRKLRWLILRGCPHLHTLPSIKTKLELEVLDLCGA<br>TSLNTIKDRKLEVNKKLQFLDFSQAQVEKLPFLPNLQNLSRLLARGSRLARLPAMTALTGLQVLDVSGSALLREIPDEPSKGNIALRIFDLSGTAI<br>TKYPPNTWNIRNLYLRGCSSLLKLPSTVPLKDLELIDLSEAAELKEITDDSFEHLSFLRLLNFSNTKIRRLPSLSNLSSLSEILLMGCSLLEELPE<br>MDGLTRLEILDLTGASSLQTIKHLNYENLRRLLLKGCRKLDKLPQLVALQKLEVLDLSGCEALTEIPDGSFEHMTQLTRLDLSKTKVNILPSLSKL<br>GNLSHLSLRDCEDLRTLPPLESLSKLEELDLSGATSLKEINAVFVEHMKLQILDFSRIRRVLLPSISKLQSLIQLKLQGCSELDTIPQLEMLTKLE<br>ILDLSETKITSLPSLIHFSNMRELLLRGLNLVELLDLKSLSRLEVLNLSETKLKVFPSEFSELPRLKHLHFPVMDGMRVEWENIKCLPEEIHLGEG<br>DMLKLAETLREKENKKLSVSMTGTKLFEICMSIPGIWEAHFKQFHFSVCSADCSKDRDTFGHREETMFREIDTQTRKFSYPYENCRSLEIRGFSDI

	PSGVKDVLSCAEFVSLIDNMSLSCLSDLGADNIKGCWVEICNGMENILTSNLAATIEILWASNMANLRSLCSLQSESFNNLKELYLDCCPMLVNVFFSSKVPESLEILQIKFCDRLETVFGDGKSAESKMNKLRKLHLLELPELKSIGAVFPSLQIFEIRECPKLQNIEDMELVGNSQSENFKVLKCLSLDSCPGLINVFSSSYKAEKLETLQIKFCDNLETVFEGNNASLTNLRTLRLWNLPELKSVGEVLLPPQLTKDFRKCPNLQLQ*
14.	**>Cluster-12591.17823;orf1 len=112 frame:1 start:1 end:336 1343980740|XP_023890030.1 putative disease resistance protein At4g19050 [Quercus suber]**<br>LDLGCCDLTSETFPSNIECLSSLETLDLSGNNFESLPTNITQLPLLTRLNLRNCWRLESIPELPSSIYINTEGCTFLGRPDRDLDEIEREYYRKQLRRRRYGEFEGGCEEY*
15.	**>Cluster-12591.16531;orf1 len=240 frame:1 start:1 end:720 1389545355|XP_024923391.1 probable disease resistance protein At4g27220 [Ziziphus jujuba]**<br>LYPEDYEIERNELIACLIAEDVIEEVGSRKDAFDKGHTILDELENACLLESSGDTNWPCVKMHDVIRDMALQIRGRESMVECGVQVVDFPNEERWKDNLETVSLMGTNIKEIHVDASPRCSKLKTFLLSKSRMLTSIPDSFFLHMPSLCVLDLSRTKIEILPNSISELENLTALVLRESGVRYVPSLTKLQRLRVLDLCRTSNIEQIPEGMEMLVNLQYLNIRGTGAETFPIGILPKLIR
16.	**>Cluster-12591.13866;orf1 len=509 frame:2 start:2 end:1528 1344015106|XP_023890235.1 probable disease resistance protein At4g27220 [Quercus suber]**<br>KLVAKECACLPLAIMTIGTSMKGVVDKREWRNALKQLSKSTKGIDEMKKVIEQLTFSYTRLKDEKAQRCLLYCALYPEDYKIERQKLIPLLIAEELTEGMESSRDEGFDEWNTILNKLENACLLESYTYLYHTKKYVKMHDVIRDMALQIMGGKSMVEAGVQLSEFPSEEKWTEVLETVSLMDNNIEEIPIDASPRCSKLKTLQLSESRSLKMIPDSFFVHMPSLSVLDLSYSAIEILPNSISDLENLTALMLRRCRNLYYVPSLAKLRRLRMLDLCGTKIEEVPEGMQMLEKLQYLNLFIDCLKTFPTGILPKLTRLQCLILYCGSKTLYIKGEELIGLKKLERLECKFYDLNGFNLYVRSLEHHQRRLPKSYDLYVGSVALCDPFYDDDDDGKHKKKVRCGIRREGEAESKSEESILVLHNDVEDLRIQYCDDVRSLSDVRVQLLSNLEVLRIFGLKNLCAASSHSPLLLHCTFSCLRELHIVDCRNIKRLFTPGLVPHLQNLEEM*
17.	**>Cluster-12591.13864;orf2 len=119 frame:2 start:2 end:358 1173804601|XP_012065341.2 LOW QUALITY PROTEIN: probable disease resistance protein At4g27220 [Jatropha curcas]**<br>WEAFNLESVGIPEPTIGNGCKLVLTTRSLDVCKRMNSLCIKMELLSNDEAWNLFLERVGRDLSMSPDLEAIAKLVAKECACLPLAIMTIGTSMKGVVDKCDWRNALKKLREPTRGSMK*
18.	**>Cluster-12591.13853;orf1 len=260 frame:3 start:3 end:782 1344015106|XP_023890235.1 probable disease resistance protein At4g27220 [Quercus suber]**<br>KCVKMHDVIRDMALQIMGDKSMVEARVRLGEFPSKEKWTEVLETVSLMNNNIEEIPIDASPRCSKLTTLRLSKSKSLQMIPDSFFVHMPSLSVLDLSDSVIEFLPNSISDLENLTTLMLKRCANLYYVPSLAKLRRLRMLDLCTTCIKEVPEGMEMLANLQYLNLYIIHLKTFPTGILPKLTRLQCLILFCGSETLNIKGEELIGLKKLERLVCQFYDPNDFNLYVRFLEHHHRRLPKSYDLYVGLISLRCDPFYDDDDG
19.	**>Cluster-12591.11042;orf2 len=72 frame:2 start:1994 end:2209 1173804601|XP_012065341.2 LOW QUALITY PROTEIN: probable disease resistance protein At4g27220 [Jatropha curcas]**<br>FFSWLWYMQFRGKLYRKGRWKNLGPFIHKVRVYGMGGSGKTTIMKHIHNPLLGESGKFDNVQLDHCIRLLV*
20.	**>Cluster-12591.23219;orf1 len=336 frame:2 start:2 end:1009 731403515|XP_010655095.1 PREDICTED: probable disease resistance protein At5g63020 isoform X1 [Vitis vinifera]**<br>LWIGGECGAMKDRFFVQAGVGLTETPSNVKWEEVEKMSLMNNKIVELNGTPECPNLVTLFLNNNYLEMITEAFFQSMPTLRVLDLSRNSINRLPKENCEVVSLQYLNLSNTRLKELPAEFKNLVNLKYLDMSGSEIHIIPKGIFPNLRKLQVLYMEFEYYPPSDVGETLVKELESLKYLNCLSISIDSSSML

	SDFSCSPKLPIITTGLRLKHFKKSKYIDVSFLQNMTSLYRLSIYDCFELEELFIQWEGKEVIPKSIGHNCCFLSLRSASINNCPTLKDLTWLTFAP<br>SLSNIHIVSCCSMKALIGEWKWGGGDAGHGAGGGGTTQCRIFSKLESL
21.	**>Cluster-12591.16819;orf1 len=107 frame:-2 start:470 end:150 1050637815|XP_017642004.1 PREDICTED: probable disease resistance protein At5g63020 [Gossypium arboreum]**<br>KEAAIYAGHGGGGGGTTQCCLFSKLERLYLWNLRNLESIYSNALPFPCLKQLNISLCPKLKKLPFDSNTAKGFVIEGSNEEWWNRLEWEDEATKEC<br>FSSSFHAIPY*
22.	**>Cluster-12591.19837;orf1 len=148 frame:2 start:5 end:448 1585766578|XP_028078155.1 probable disease resistance protein At5g66900 [Camellia sinensis]**<br>KWEADTLPLSGKFKDKILFVPVSKTPDLKVIIQRLIEQKGPVPNFLNDEDAVHHLEQLVKRLGPGPVLLVLDDVWSGSESLLEKFLFPIPDYKILI<br>TSRFKFPKFDSTYELGILKDEDAMKLFRYSTFPQDGSAYTPDEDLVKKVFI*
23.	**>Cluster-12591.33141;orf1 len=923 frame:-1 start:2939 end:171 359496974|XP_002264069.2 PREDICTED: probable disease resistance RPP8-like protein 4 [Vitis vinifera]**<br>LYQNFHKVFWQEVSKNTPEMAEALITFFLENLAEQLVQEGKLLSGVQQDIEWIKSELQAMVAFIKAADRRQQRRNDDESIRNWVGEVRHLLYDAED<br>IIDEFVIQMGAIRWNNIIKQYQVRRRTSCQIKKIKSKVVEVKERRDRYGFHLQDDDAATSIVRGSSRGGPGAASPFVQEDGIVGIEEDVEQVRKLL<br>LDGKMGCGRLVISVFGMGGLGKTTLVKEVYKKCKTRFDCYSWVFVSQFCSLRDLLRSILFGFKASRGEPAMEVMDAMDEGQLQERTYHYLQDKKYL<br>LVLDDIWDAKVWEELKHALPRETGRIIFTTRIRGIASPVEDNCRVYDLQPLSNELAWSIFCRKAFRGKLVCPEELKEFAEAIVRRCGGLPLAIVAI<br>GGLLSSRNANLPEWQSILHTLDWEVNHNRDFERLNTALLFSYNHLPFYLKYCFLYTGLFPEDYEIGRKRLIRMWVAEGFVRKTSSKTEEEVANHYF<br>TQLIDRSMIQAVMLHARDVVKACRVHDLMRDVATDMLKQEKFGVVLTDGDSSIEERQRRLSIYDSIENVPSNMGKLNLHSFLMFRLPELSPSAFRK<br>LLLELVLARVLDLQGVPIDILPDEVGNLIHLRYLDLRGTQIERLPKSLKNLRNLQTLDVRNTNIRSLPPGINMLQQLRHLHMASFRDRDKGLLKMP<br>KGRKCFKNIQTLSGVECDDELVKELRDLTNLRKLYIGGVTGANSKELFASIAEMKNLRSLTIISDESSLEQHQIESLSRAPLYMEKLKLQLSMRRL<br>PKWFDSLKCLHTLYLFKNFLQEDPFPILERLPNLVILTLASSAFACEAICCSKQGFPKLKLLRILDIANWKTWKPIEEGTMPSLQYLLIADCPKLR<br>QLPEGFHHLTALQDLTLKGMSTHFSYNLKGSGRWKVSHVEEVSILTQVGGEFVRRKLE*
24.	**>Cluster-12591.15434;orf1 len=848 frame:-3 start:3276 end:733 1098788817|XP_018855747.1 PREDICTED: putative disease resistance RPP13-like protein 2 [Juglans regia]**<br>IQRYAASKEHTEPVSTWLDGAKKLVFETEDILDTFISWKEQRRKQKYTFFIQFRNNFSNKLKRIQSKYCTSSQDRLRYGGINPPEDGDETSSFIID<br>RRAETDSLVKEYDWFFREAEAIGLKDDVNLLVSRLIDEEEDVQKQHRRVISIVGMGGLGKTTLAKLLYNHSRVIEYFEIRCWIYVSQDYRVEEIVN<br>RIWNSITGHRESSLTVEQSTARLCKFLDGKKYLFVMDDVWDADAWLKIEALLPDTGNGNRVLLTTRARDVASRVDPRSLTHHLKFMSEEDSRTLLR<br>RWVKLSGRGTIHTINSDFSREIIAKCGSLPLVIKTVAGLLWMRNKDEWYNLLNRFFEFSNYTDLDFFKRMLELSYNDLPSHLKPLYLNLLFFPKEH<br>DIPLRRIFGLWLAQGFLTGNHEEVSREELARGYLYELIVRNMIEVVKWKLDGTPKTFRVPGRLRDELLPRATDIGFFHNQNINSDTAPSGRTDMEA<br>KIPASVPDAPATPKYVRHFVEYTDINDSISLERFIRNSRSYISFNFRIGDSPAEVTGRFLGRVIQKHSFRLLGILDLERVYKPKLPDSLGKLHLLK<br>YLGLRWTFLDAIPSSVGDLLNLETLDVKHTYISRLPSSIRKMKKLQHLYLNEIHLDLSVGDIALQNLQTLWGLTADGENPIENNLNCLITLTKLGL<br>KVQDTLVEVYAKWILRLTALRILRFRSRDECGRPSDLKLGSFSSLSRLYDLYLLGKLTMENPLHELQLPESLTVLTLSVSGLEQDPMPTLEKLPNL<br>RALRLFANSFTGKRMVCSNGGFPSLCILKLWMLVELEEWIIEEEAMSSLEQLEIRCCKKLKKCPKNMLQLENVPRICCS*
25.	**>Cluster-12591.10530;orf2 len=1414 frame:-1 start:5247 end:1006 1216238872|XP_021600003.1 putative disease resistance RPP13-like protein 1 [Manihot esculenta]**<br>LINGNANGNGVKKFHNHCVWIVDHHFHSSHKPCGLHHFYQSLLLTTRLSSCLCVSVISTLFRFHFDLPFSFPSLSELSKSTSFMEAVGEALLSAVL<br>GVLLDRLASNHVLSFIRGRDIEKELKGLKKTLVKVNAVLNDAENKQFKNGLVKEWLDELRDAAYDAEDVLDEFATEALRMKLEAESQTDSNQSWSL

	IPASLDLSARFFNAQVQSKIKAITLRFDELAQERFELGLEEFASGSSSSNITRETSSVVDESCVYGRDDDRETIMKLLIPEVGPSCTINVFSVISI<br>VGMGGVGKTTLAQILYNDERVEMHFDVKAWVCVSEEFDVKIITKIILESVSPGTYDFSDLNQVQVKLKKALTGKRFLIVLDDVWNKKYSYWDLLRK<br>PFNVGAAGSRIIVTTRNREVASKMDTVYDHYLKHLSEDDCWLVFVHHAFGSKRLNANPTLAAIGKKIVTKCQGLPLAARTLGGLLCSKQEEEEWEN<br>VLSSKIWDLPEEENDILPALKLSYHHLPSHLKRCFAYCSIFSKDYEFGESEVVLLWMAEGFLQQPEGKKELEDLGHDYFRELLSRSFFQPSTSNKS<br>LFVMHDLMSDLALWVAGKICFRLEDLLESCEILKQARHSSYIPSVSDGVKRVEAFYECVCLRTLLPLNTTRWCRLTNDSTIELLPKLRRLRVLSFY<br>KTQISELPGSIGDLKHLRYLDLSFTPIESLPDSVCTLYNLQALILEFCMHLKKLPTKMGNLINLRHLDITYANLIEEMPPRVGRLTGLRKLSDFVV<br>GKDVGSGIGELMNLNHLQGRLRISKLENVTDAQQARDANLNDKKGLAVLEMKWCSSQDNRNERVEKEVLDMLCPHEKLEKLTICGYGGVRFPTWMG<br>DPSFSNMVQLRLEDCVKCTSLPPLGQLSSLKDLYIKRIIGVKVVDLEFYGKGSSIPFPSLENLSFEDMQEWKDWFPGEIDKQVEVFPRLQKLSIMS<br>CHKLLGSLPHHLPSLETLTVHRCELLTVTISNLPRLCELEIGNCKEVVQRSKVDLSSLKSMDLWCISKATCLTGGVIKGLTGVEVLSIVDCDELQK<br>EELQNFASLRRLKLISCKNLEDLQGLHTLSFLTKLSIEACPRLVSFPERSLPSMLEALSIRNCSALKSIAEAILWHNCTSTSLKRLYIERCDNLMF<br>IGRGPLFPAVKVMQIESCRSLQCLLDGDEIADSSSSSSSLCCMRAQDSHESYRDLLQSLLINNCPSLMSLSSSSTTGELPATLNHLEISECKSLTS<br>LSPRGELPLKLKHLQIWECLKLESVGEGSQGSMPVELESIQIGRCRNIFSKSFPHGLHRLRTLEIYNCDNFVVSFPEGGLPVTLESLCIVGCPKLE<br>SIGERFHNNLSLESMVIQNCGNLKSLPEGLHNLTGLTVFEISKCPSLVHFPDGGLVPCHQLGKALDFRL*
26.	**>Cluster-12591.3030;orf1 len=891 frame:2 start:125 end:2797 225445258|XP_002281054.1 PREDICTED: putative disease resistance RPP13-like protein 3 [Vitis vinifera]**<br>GVVLQEKEDMAEIALAAVIQKSIDILGNLVVEEGTRFYWLREHVLWIEAEMRRIQSFLKDADAKKTESQLVANWVREVRDLAYDVEDVMETFLHKL<br>IRKGQLSERLTWFYPFGSSVSKFVMEIEVIKRRVEDVNHGCRNYGIVGISGSGGVKEEWNQRRSFPHVDEVNVVGFEKHIEEMVTKMIDESVRFCV<br>VSIVGMPGLGKTTLAKKVFNSVKRNFECSAWVYVSQEPRVRDLVQDIARQVGLGKEKYDENIEANLFEFLKNQRYVIVLDDIWKISAWDALKNVIP<br>HDGKKGSRLLLTSRIRDVGVYASGDHCLHSLKPLTPDLSWQLFSKIMFSTRETNGATCTKELKELGGQIVKRCGGVPLAIVVTAGQLLQRERTEPA<br>WRALLKKMGQEQGLVSGILALSYVELPSYLKPCFLYFSLFPEDHEISVSELVNLWVAEGFIQANGEQTLEDAGEDYLNELINRNLIQVSRRRYDER<br>IQTCRIHDLLHNLCISIAKEINFLTSLKDVDSLSAMRVRRVAIYNNSNNHDFSDHQTPKLRSLLFFNQGDDDGLKRRRYKRMDKRFRLLRVLSLEG<br>EVTFPQTLTDEICNLIHLRYLKLRGPNLQRLPSTISNLQNLQTLDARECKYLILPTSILKMKQLRHILLYGKAKFKHPVKLGNIMDRFQPVDEVCL<br>PNLQTLSMVHGGCLKAACLREFKNIKKLGIRYSIRRFGEVLTGATPISEKLESLKLDGLYYPYKVRRLHNHFSHRPVNPALDLSRYRYLCKLYLKG<br>TLQRLPNPNEFPPNLTKLTLLSTFLKQDPMETLKQLPRLKILKLGFDSYNGRKMVCYGASGFPLLQVLELADLPYLEELKVEEGGMPRLEQLRIYE<br>CHKLKKLLNGLRSIRTELKRKVEYGK*
27.	**>Cluster-12591.23115;orf1 len=518 frame:1 start:1 end:1554 1365947340|XP_024175248.1 putative disease resistance RPP13-like protein 1 isoform X2 [Rosa chinensis]**<br>VLLSLSLSLESHHSTMPFTRALESASINVLLNKLANQDVIDFFLKWKIDGGLLTKLKTYLLMVNALLNHAEERQVEDPHVKAWIDMVKDATYDAED<br>ILDEIATDALESLNSTSKVRKSIYDSVSASESVKAGIDFKMKDIVSAINPFKERIESKMTSIIERLKDIAKQKDGLRLRADDGGKSVRVNERLTTP<br>MVDESHVYGRARDKETIINLLISGEGHGDEVGVIPIVGMGGVGKTTLAQIVYNDDKKVKGYFHQRAWACVSDEFDVTRITKALVVSATKSRSETKD<br>LELLQEELKNRLEGKRFLLVLDDVWNEDYTSWDALRVPFTVGLPGSRIIVTTRNNKVAKIMGSFPIYDLKELSTGECSLIFEKIAFPNGNPDAYPK<br>LRKRGKKIVQKCKGLPLAAKALGGLLCSETDEKYWKDILHSKLWNLPNDSILPALRLSYHHLPSHLKQCFAYCSMFPKDYEFQKEKLVQLWMAEVS<br>CSSQIEMKDWKIWAENIFLFSYPGHSFNNIVAINQDM*
28.	**>Cluster-12591.12024;orf1 len=932 frame:1 start:160 end:2955 1375836295|XP_024442377.1 disease resistance protein RPM1 [Populus trichocarpa]**<br>GIQNRKMAESAVAFLLNKVAAILQEEGNLLKGLQGEVGWIRDELERMRVFLRVAADMEDDDEELKVWVKQVRDVAYDTEDALDEFMLRLAQNPGEG<br>LRFAAHKAFSPVKNLRARHQMASKIRSIKSRINTISDGHQRYRCKFDVVGQEISNSFSGNCGWYDSRGDALLIEEAKLVGIDKPKRQLIGWLLEGK

	SGLNVVSVLGMGGLGKTTLVKRVYDSAQVKKHFQAHAWINVSQLFNLEELLKDIMRQLFIEISQPIPEGVESMNINQLKAIINEFLRERRYVIVLD<br>DVWSINNWDAFKYALPNSSCGSQVLITTRIEEVAFGASIDTQGHAYTLKPLSLQESWTLFCRKTFQKDSCPPHLVKLSRRILKRCEGLPLAIVVIS<br>GVLATKDSRIDEWEMINRSLGVELATNDRFKSMNKILSLSYSDLPYYLKSCFLYLSIFPEDYLIERMRLIRLWTAQGFVEEKQGKTVEEVSDGYLN<br>ELLNRSLIQVARTTTDGRIKLCRIHDLMREIILAKSKDQNFVAIANEQNTSWPEKVRCLSIHNSFENVQQSKRVAQLRSLVMFQVTGSFFNSSMFA<br>MFGTDLKLLNILDLRGAPLEVFPEEIVELFHLKYLSLRDTKVRRLPNSIGKLRNLETLDLKRAPITELPIEILNLQRLRHLLLYHYDESFKVGFPP<br>SINGFEAPSGIGELSSLQRLNHVEANHGGSLVREIGTLYQLRRLGIIKLRKEDGEALCSSIEKLCNLRSLSLESIEEEEIDLQSLSSPPQFLRSLE<br>LGGRLEKLPYWLPSVHSLVKVILRQSRLNKDPLESLGALPNLVELGLFQAYDGETLCFKAGGFQRLKVLWLRTLQGLRMVRVEAAAMPKLERLCIW<br>SCKMLQRVPSGIVCLTNLNYLSFYDMPDELMETLKPNKRAGDYWKVSHISNVYIGYIRNGRLTGYRL*
29.	**>Cluster-12591.17963;orf1 len=962 frame:-1 start:3492 end:607 1389646767|XP_024927732.1 disease resistance protein RPM1-like [Ziziphus jujuba]**<br>ARYREKQLPKCKHKEREMAETVVNLVIENLLALLYEEAELLKGVHKDIRDIKDELESIASFLKDADARAEMGDNNNNNDGVKVWVKQVRDGAYSIE<br>DVIDSYMLHVAQHQYPNRFIKFLRGVTQSSKNLKTRHQIATEVREIKGSIKEIYDRSIRYGFNSSSSATTHQGSRATWREQKAAFPFVEESQLVGI<br>EAPRDELISWLDKAVLKRAVISVVGMGGMGKTTLAKIAFDKRTKTRGHFDCHAYVIVSSSYKNEDLLRIMMKQFYETSGELVPNELIDAMELSVLI<br>SKTREYLQQLRYVVLLDDVWQIDFWKFIRHVLPDNDRGSRIIITTQHQNVASSCKECPFDYLYKMQPLLEENAWQLFCNKAFQSEFEGHCPPGLRD<br>LSLGIVKKCEGLPLAIEAMGSLLSTKEKDVSEWRKLHDDLGTELESNKHMSDVKNILYISYSDLPYYLKPCFLYFGMFPEDEFINCKRLIKLWIAE<br>GFVKRMGDKTLEMVGEEYLTELIHRNLVQVKTVNFLGKVKRCRAHGLMREIILIKSQDLSFCQILAKDGSCIEGRSRRLSIFYNKENATKNISNSL<br>IRSVFLFRMNTLPNTYLTTFCANFKLLKLFDLQYAPLKFLPKEVGDLLNLRYLSIRGTKVETLPKSIGKLRNLVTLDFKFTRVQELPIEISMLHKL<br>QYILSFFKNGEADPSFNSALGVKIHKGIGCLEALEKLYHVEANQGIDLINELGNLRKLRKLGITKLTTTNCQALCSSIEKMIHLRSLDVCSLNPDE<br>IIDLSSLASPPPFVQHIKLCGRIATLPDWIPMLQELVILTLGWSGLSSDPLQSLQGLHNLQKLWLRKAYDGDQLHFKAGWFQKLKVLGLIHLNGLK<br>LLSIDNGAMPHLQELTLGPSLQLKEMPSGIQHLGHLTALQIFDMPRDFVLRTLPEEGVDFFKVKHIPAVLINRMMEEGGYHTYTLSSDSTEIKHCY<br>K*
30.	**>Cluster-12591.33361;orf1 len=940 frame:3 start:126 end:2945 224143387|XP_002324939.1 disease resistance protein RPM1 [Populus trichocarpa]**<br>EREREREGEFTDQRNNMAMVAVEMVLEKLTSLISEEAQLLGGLPKGIEELRDDLDSMKSFLQDAEARSENDHGLNTWVKQVRDVAYDTEDVLEEFL<br>IRFSLPHHHGFIHTLRNCYLYIRQLRSRHRLAVQMQHVKQRVRSISERRAAFSLNITRSDEATSSNATVCTRHDPRLASLYIDEADIVGIEKPKNL<br>LIKWMVEGEEKLTAISVVGMGGLGKTTLVKKAFDSQVVKRCFDCHAWVTVSKSFAVAELLQAILKGILEATKEPAPEALDQMKDLQLVDALRNHLQ<br>QKRYVIVFDDVWNINAWEAVKYALPDCNCKSRIVFTTRSTDLAASMETTCHVYHLQPLPEQEAWVLFCTKAFRREHKCACPKELEGMSCSILKKCG<br>GLPLAIVLVGGVLSKKNENVLEWKRIHDSLAAEMRSDYNLESLGRIILLSYDDLPYNLKSCYLYVSVFPEDYLIKRMKLVRLWVVERFVQEKPGLT<br>LEEVAEEYLNELVSRSMIQVVDMDFFNRVQTCRIHDIVREIIQQKSKDESLVTIVNERSMKKNDKVRRLAVHENCEDFPSYTRFPHLRSLLVFTSK<br>GSSITLPNACFHGFKFLRLLELELAPLSQFPLELVELIHLRYLSLRRTMIRELPDSIKKLQNLETLDLKGTHIPSLPVGILQLKHLRQIRNYYFRF<br>GTSLFFPDSYGMRVPLGIGRLTNLEKLGSVEVYDKPEIVRELGNLTQLRRLGILKIRKEDGIEVCSTLQKLRQLTGFYMVSIGTDESVDLNSLVSP<br>PEFLQRLYLKCPLPNLPKWIASLQYLSKLVLQYSNLKSDPLNALQGLTSLMVLELRGAYDGEELCCEAKGYPRLKKLSLYELQNLKCIRVEEGAMP<br>ELRRLDIASCEELETMPLGIENLKNLQNLLLWDMPSPFLEKIKQLSGEDYWKVQHIQTITRIYESRGTWAVETIS*
31.	**>Cluster-12591.27834;orf1 len=936 frame:1 start:58 end:2865 694398767|XP_009374540.1 PREDICTED: disease resistance protein RPM1-like isoform X1 [Pyrus x bretschneideri]**<br>LSPLKPFSNQASMSEIIVRHVFQNFVTLLIDEPGVFKPHLRKEILKLKDDLDAISTVLKDADEQASDNYSVKSWVKDINEVAYLLEDAIDGYMLYV<br>AQHRKQHKLLSSFHLFGGEVLKGNEVALQIQKMRELVDRGQYFRFSSSGAEGPSDSLSSVESRLTEDSLFIEETELVGIESSRDQLISLLLMKEGA

	SKPKVISVVGVGGVGKTTLVQTVYCSPTAKGSFECYAWINVSHSYKLKDVLCVMILQLGMAGKESAPSDMEFMEVALLMKTLWEYLQRKRYLVVFD<br>DVWNTDFLESIQYALPDDNNGSRVIITTRNSMVASSCQTSFDNYVFWMEPLSEEEAWKLFCMKAFPYYLGGQCPPELRELSLRIVRKCRGLPILTI<br>LVGGLLSTQEKTISQWQKIYKDFEKAGERSMLDTGILPFFSLSYNVLPYNLKSCFLYLGIFPTNYSINCSRLIKLWIAEGFVEKKISMTLEEVAEE<br>YLSELLRRCLVEVTETNYCGKPKNVRVHDLVGDFISFKSMELSFSHVLIDNEHSWVSRRVRRLSIQYNKQGNMPRNIMHSLIRSVFLFKVDSFPII<br>FFTEFRDEFKFLKLLDFQDAPLKYVPKEVTSFLHLIYLSFRGTNIKELPRSIGKLNNLQTLDLKFTLVSELPPQIKKLLKLRHLLVSNPFGGVKIE<br>EGVGHLEALQKLYYVEADHGVELIRELGKLRQLRKLGIKKLRTEHCGDLCSSIEKMNHLQSFYVHALSEDEIIDLQDISSPPRYLRHICLEGRLSK<br>LPNWIAALHNLARLHLTSSRLIEDPLTILKDLPDLLWLGLYHAYDGEQLYFESEGFKKLKMLELTNLKELSMLKVDEGALPLLEKLTIGHSLELKE<br>VPSGIQHLKNLTTLTFVDMPKPFVHSMLPDEGRAYSKVEHISTVLFKQKTNGAYQHTYSLSNPEKLKQSYN*
32.	**>Cluster-12591.15032;orf1 len=159 frame:2 start:104 end:580 1552262914|RVX06979.1 Disease resistance-like protein DSC1 [Vitis vinifera]**<br>TLQYISTESFRSWILEISITFPSFFGLSALTVLNLSCCNIRDETFPSNIECLSSLETLDLSRNNFESLPTNITQLPLLTQLHLSFCERLKSIPELP<br>SRIRIHTYGSWSLSGRNLALEMENRLREQMQRQREQWAAQSCGKFQCCFLTLLCLVKASRKI*
33.	**>Cluster-12591.17824;orf1 len=164 frame:-2 start:493 end:2 1389637329|XP_024955230.1 disease resistance-like protein DSC1 [Citrus sinensis]**<br>EKLPENIGDLESLVELQAFSTAIKEVPASIQFLDKLRNLSFAFCRGLKLPSFYGLPSSLTELDLSGCDLTDETFPSNIECLSSLETLNFSFNKIVS<br>LPTNITQLSLLTRLYFRGCERIKSIPELPSVFIDTFGCTSLTSISTPTISKSGLSLSLSLSLYIYIYR
34.	**>Cluster-12591.13408;orf1 len=146 frame:2 start:2 end:439 985431198|XP_015387893.1 disease resistance-like protein DSC1 isoform X1 [Citrus sinensis]**<br>VRDMGRHIVHQESPKNSGKQSRLWNIEDIRRVLEKNMGSKAIEGIQLKTRMFDSTFKAKVFMKMKNLRFLSISKTKIQFPEGFSFPDKLKILVWNY<br>YLCEHSALDFLPDKLVHLSLIVNSLKLLWNGRKVRTHVTCLLCLSLLFP*
35.	**>Cluster-12591.26342;orf1 len=345 frame:-2 start:4328 end:3294 1585782181|XP_028085714.1 disease resistance protein TAO1-like [Camellia sinensis]**<br>MYNSMAPEFWDFMGWVGLVRQPLPRLFIINLSVIFIVVVSFQMFEKLGHMKMVVSLQNKLMCDLSGVKVVPALNEVSAGVNAIKEIVRGRRVLIVL<br>DDVGDISQLDDLAVRREWFYEGSRIIITTRDKNVLLQQHVNDDYEVRELDSSQALQLFSYHALRREKPTENFLNISEKIVSLTGGLPLALEVFGSF<br>LFDKRRLEEWKDAVQKLERCRPSNLQDVLKISFDGLDEQQKCIFLDIACLFVQMNMKREDAIDILKGCGFMAEIAIMILTTKSLIKIAEGKLWMHD<br>QLRDMGRQIVRHESLLDPGMRSRLWNCDEIMTVLNNKKVWIISVPIVFLNDIEDFM*
36.	**>Cluster-12591.13240;orf1 len=728 frame:-2 start:2184 end:1 1585749361|XP_028070380.1 disease resistance protein TAO1-like [Camellia sinensis]**<br>CFAFTATKTWQVVVRSGAASKQANMADDDDTASTDSTPSFRYRWDVFLSFRGETRHTITENLYNALLQNGVRTFRDNEGLNRGDEINSNLLEAIED<br>SAASIAIISEGYASSRWCLQELTKIIECRRLLLPVFYQVDPSDVRRQKGPFEKDFRILEERFGVEHVRPWRRALEKAGGIAGWYSPIWDEQTLIDS<br>LVKEVLNKLSNTPLGVAKYPTGLNSQLEEVMKKLDVKANGVRVLGLHGMGGVGKTTLAKAVFNKLVIHFRRRSFISSVSEIWKNHNGSTSLQSKLI<br>GDLSSSAIKIGDVSEGILSIKKLIDREEPILVVLDDVDDVEQLHALACRRDWFYEGSRFIITTRDKEVLHESFVNELHEVNELNPSESLQLFSYHA<br>FGRDKPAKDFMNLSKQIVSLAGGLPLALEVFGSFLFDKRRRKEWDDALRKLERIPPGRLQDVLEISFNELDEQEKCIFLDIACFFVKMNMKREDAI<br>DIFRGCDFNAEIAIKVLTSRSLIKIIENDTLWMHDLLRDMGRQIVRRENNGDPGMRSRLWECGEILTALKNEKGTNTIQGIVLDFEKKHDLSSEEI<br>CWNNFKKQPGFSSAVTYLKEIYKTWFHHGAENGEDVMLLTKAFKPILNLRLLQINWMKLSGSFRFMPAGVKWLQWKGCFLKNLPYEFCLGDLAVLD<br>LSESKITQLWGWRWWVWSQKKMAEKLLVLNLHDCRYLTTIPDLSGHLVLEKLILEN

37.	**>Cluster-12591.15397;orf1 len=224 frame:-2 start:674 end:3 508721744|EOY13641.1 LRR and NB-ARC domains-containing disease resistance protein, putative [Theobroma cacao]** TALKELRECTKGFKGMSKVIEQLKYSYTRLNDEQAQRSLLYCALYPEDYKIERNELIAGLIAEEIIEEMESRKDAFDKGHAILDELVNACLLESCSQFVGSDKMNCVKMHDVIRDMALQIRGCESMVECGVQVVEFPEAKRWKDNFETISLMGTNIKEIHVDSSPRCSKLKILRLSESRMLTSIPDSFFLHMPNLCILDLSHTGIEILPNSISELENLTTLVLR
38.	**>Cluster-12591.22764;orf1 len=279 frame:2 start:2 end:838 508775941|EOY23197.1 Disease resistance family protein / LRR family protein, putative [Theobroma cacao]** VIPESLGQLSNLESIYLGSNFLEGALSEIHFSNLSKLGTLDISFNHLSFNVKPEWVPPFQLSYVGMSSCKLRTPFPQWLATQKEVSSLYLANANISGPLPNWLWSMQFHRLDLSHNRITGSLPQNIGDMVPMLESFYVADNLINGTIPISFCKIDTLKFLDLSNNMVSGDIPNCWSSSNQLNALKISFNKLTGVIPNSIGHLIELEWLHLKNNRLGGEVPLSLRNCRSLKLLNLAQNIFSGNIPTWIGESLLSLQILRLQKNMFDGYIPRQLCKLTTLK
39.	**>Cluster-12591.17830;orf1 len=138 frame:2 start:2 end:415 1335245797|PNY04749.1 disease resistance protein (TIR-NBS-LRR class) [Trifolium pratense]** RDMGRHIVHHESPKDPGKRSRLWNTEDIRRVLEKNMATEAIEGIQPETFMYDWSFKDEAFIEMKNLRFFGWPYYRSEHLAFDFPHDKLVHLSLIGSTIKLLWNGRKVLPHLKSIDLTWCVYLREFPDVTGLPNLERLS
40.	**>Cluster-12591.31056;orf1 len=455 frame:-2 start:1365 end:1 1371522980|PSS19074.1 Disease resistance protein [Actinidia chinensis var. chinensis]** QGELQRSVISIFGMGGLGKTTLARKLYNNDAIKSRFDQHAWVCASQEWTTRDILERIVKSFKNHSRTELENIEKMNAEDLERYARELLEGRRYFVVIDDVWEIAVWETLRRAFPDNKNGSRIIITTRIEAVAEFSGQRTYVHKLPFLQPDESWELFCKKAFPSGETSFAGGMEQLGREMVAKCGGLPLAIVVLGGLLSRKELHEWPAVKDHIWRELKNNSLHISALLSLSYSDLPSYLKPCFLYLSLFPEDCVFNKERLIRLWIAEGFISQREETLDLEQQAEEYVKELIGRSLIQVVQGNWRSKIATCRVHDLLRDLAVSKAKELNFLHNYDENNDFTTTNRRRLAFHSELKRGVRLDKYDDHIRTLSFHPLSISEEFVSLSIDELTRFRLLTVLNLGELSISSSYLNEIGKLIHLRYLGIWASEALALPKTFANLQALQTLEFFRTSRYVELP
41.	**>Cluster-12591.17357;orf1 len=127 frame:-3 start:382 end:2 1552215560|RVW67961.1 putative disease resistance protein [Vitis vinifera]** KWEEVERMSLMQNKIVELTGTPECPNLLTLFLNNNGNLEITDAFFQYMPTLRVLDLSNNNSIKRLPKEICELVSLQYLNLSSAKLKELPIEFKNLVNLKYLNLSWSAIDIIPEGIVANLIKLQELYM
42.	**>Cluster-12591.32027;orf1 len=901 frame:3 start:129 end:2831 1552270775|RVX13547.1 putative disease resistance protein [Vitis vinifera]** LCHRLLKPKMGNLFSVSISTDSVASCCWICGKQAMHGWKLGENIHLLKDDWDELKRMRNDLLRRVYNEEGPQMKRLDQVEGWLLKVEGMEDEVKKLMINGNREIEMTCLSGCCPKNCKARYKLGKKVAQKLKEVADLRRKGHFDSVVGRLASPPAEARHCEPTIGIESMIGKVWEYIRDDQVGVIGLYGMGGVGKTTLLTEINNKFSNTSHDFNFVIWVVVSKNPSLEKMQEEIGKKIGYGDKMWANKSQIQKAEDIFKVLWGKKFVLLLDDIWKRIDLKQVGVPFPNEENKCKIVFTTRSEDVCGHMEAQKEVKVNCLAPDEAWDLFKMKVREDTLSADSEIPKLAKLMAQKCAGLPLALITIGRGMASKRRPREWSLAVRELEKNIAMFSGMEDEVFAILKFSYDILPSDGVRSCFLYCSLFPEDYYIPKKKIIGYWFGEGFLNEYDMLLGHNIIDTLVHACLLEEEDNDGVRMHDVIRDMALWIACEHGAAKKKYLVRPRAGLTRALDAEEWNGAKRISLASNHIEILPEAPSCPNLLTLFLSENRLKMIPSGFTRCMRALTVLDLSHNEGLIELPYDIVFLSSLQYLNLSGTSIDRLPYALKELVDLKYLDITNTMNSVRRFPDGLISRLLKLQVLKMFGWGSSDKVLQELEKLNDLKDMSLTIGNYSSALREYLISNIRVLRCITRLGFSYLQDASQVDLSFLLERMERLDRLEITYCEDLQQLNFKGVRSHPCFGSLRSVFVVRCQAITDLTILSCAPNLKLVLIAYCNGIEEIIDENSGVAWEIFSIQKELYLIELPKLRSIYAKVLPFPCLKFIRVEGCPLLKELPLDSQTAKECELTIQGDEDWWNGLNVKVEASFILFDENEKKKEKAESSSSSSNPTADAYLPYFKSFD*

43.	**>Cluster-12591.18102;orf1 len=112 frame:1 start:1 end:336 852445295|AKN63629.1 disease resistance protein N-like protein, partial [Nicotiana knightiana]**<br>CKLKYLEELHLQGCGNLDKLPENIGELESLVELDACCTAIKEVPASIQFFVKLRKLYFGYCRGLKLPSFYGLPSSLTVLDLSRCDLTDETFPSNIE<br>CLSSLITLYLSGNNFV
44.	**>Cluster-12591.31057;orf1 len=604 frame:3 start:387 end:2198 1371522981|PSS19075.1 Disease resistance protein [Actinidia chinensis var. chinensis]**<br>KDMHARLERYAREILEGCRYLVVIDDVWHTQVWETLRRAFPDNKNGSRIIITTRIETVAEFSDQRTYVHKLPFLQPNDSWELFCKKAFPTGGETSF<br>AGGMEQLGREMVAKCGGLPLAIVVLGGLLSRKELHEWPTVKDHIWRELKNNSLHISALLTLSYDDLPSYLKPCFLYLSLFPEDFVFNKERLIRLWI<br>AEGFISQGEETLDLEEVAEEYVKELVGRSLIQVVQGNWRSKIATCRVHDLLRDLAVNKARELKFLHIYDENTDFTTTTTYKRRLAFHSKPKRCVSI<br>DKLDYHVRTLSLHPFSISEEFLSKDQLTRFRLLTVLNLGELSFSSSGLNAIGKLIHLRHLGFWVSQNLVLPKTFANLQALQTLDFSTPLFSSVEIE<br>IPKWISKLKCLRHILGNVKVSPKSLQKLTNIRTLKFFRVNEFHKELGLRPIGELINLKVLDVRCYRSKCAVSIEPLLNCGHLQELTVGGNIESLPE<br>EMHKSLPNLKRLSLWNSELKQDPIPILQMFPNLVFLELNRIGSSVLEFTSQGFPQLQALIFVCSYNINEEVRIMDEGAMPKLRGISISPDCCHSLP<br>ERLSSLPPVHSLNWDWDLYHQDLDSEW*
45.	**>Cluster-12591.7244;orf1 len=917 frame:-1 start:3457 end:707 1371524701|PSS20791.1 Disease resistance protein [Actinidia chinensis var. chinensis]**<br>TFLSSLSMDAVVPLISSLIVSLYDSISTRVSHFVNLDRRIHSITSELKKLTDKRDDLKRRVDQVDSDGFMCTNQVKGWLERVEAIEVEVGLIIDDS<br>RRRRQCFWNCNVDCSSRYKLGKKIQRKLAEISELKTEADSVVIMADGSLPPPVENIPNRPTVGLEVMLEKVQQVLRNDEVGSIGIYGMGGAGKTTL<br>LKSINNEFLTNIHDFHVVIWVVVSKDFVAEKIQQAVGARLGLSWVETESQEQRAMKIHRIMMRKKFLLLLDDVWEGLDLLRIGIPLPDKKNKCKLI<br>FTTRSMDVCTDMDAHRKLKVEFLKEKESWQLFSEKVGGGMEILDSPYIRPYAKIIIRKCGGLPLALITMGRAMANKQTEEEWKHAVEVLNRSPSEL<br>RGMKAVFALLKFSFDSLDNETLRSCFMYCSLFPEDYSIEKEQLVEYWIGEGFLETSNDSNAHNKGYAVIGDLRVACLLEDGEEKTQVKMHDVVRGF<br>ALWIASDFDKNNKKYLVHASAGRTEAPDVENWVEAQRISLLDNEFTELPVIPVCPNLSTLFLQQNVALTQIHSGFFQHMPVLKVLDLSSSSISSVP<br>ASINSLVELCYLDLSGTKVRSLPKELGGLVKLQHLDLQRMYFLRVIPREAISGLTQLRVLNMYYSYGHWEVEDSNSDLDISEGDDGIGFEDLEGLK<br>KLTTLGITISEFTTLKTLSGIPTLLKCLQYLYIKDCEGLIHAQLLSGPNDGKILRRLSINNCHDLKYFVIGKNAGNRWLPSLEILTLHGLPNLVTL<br>WKTPVTRKCLRNLRSINIQHCHKLKNVSWALQLLKVEEIYLSHCDEMEEMVSDEDDVMVVGGSTEFLSLKTLEIRDLPELRSISQVALTFPSLEKI<br>VVSNCPKLKKLPFGAHRDSTLPMVYGDKEWWDALEWDDASTKAVFLPHFVST*
46.	**>Cluster-12591.21348;orf1 len=123 frame:3 start:3 end:371 1552189998|RVW49178.1 putative disease resistance protein [Vitis vinifera]**<br>GMGGVGKTTLLTKLNNQLCNSNTQGRFDVVIWTVVSKTENYKIVQDDIGEKIGFHDGMWRNKSQENKAKDIFRILSKKKFVLFLDDIWDHFDLMKV<br>GIPLPNQSNKSKVVFTTRSKDVCGWME
47.	**>Cluster-12591.12984;orf1 len=217 frame:-1 start:653 end:3 1217040495|XP_021660274.1 putative disease resistance protein RGA1 [Hevea brasiliensis]**<br>PSYSEQYRANMFLLSKFKRMRALDLRHCDFKELPKWVCKMQHLRYLDLSENKGMEVIPNSITKLVNLQRLNLSSCWNLKELPEGIGKLISLRHLVI<br>DGCNKICHMPRGLEKLTSLRTLSKFIVGGDKRRSIGIGALKDLNNLCGSLSIENLQKVRDARLESKAANLMGKPYLRELSLKWNGDDDDGQAVLAE<br>ILLEGLQPHPNLRELGIRGYRGARL
48.	**>Cluster-12591.17744;orf1 len=127 frame:3 start:56 end:437 1035902542||XP_045830474.1 putative disease resistance protein RGA1 [Trifolium pratense]**

	MPRGLGKLTSLQTLNLSNCEGLKELPEGISKLISLRYLIFDKCHQITCMPRGLGKLTSLRTLKKFVVSDDERSAKLGELKGLNNLRGDLCIENLKK VRNCGSESKEANLIGKPHLKSLTLNWGGKGF
49.	**>Cluster-12591.23128;orf1 len=872 frame:-1 start:2617 end:2 1216929794|XP_021683312.1 putative disease resistance protein RGA1 [Hevea brasiliensis]** LHYSSNHNLRFLSLFFVHLTSFFLPFYCTYNFFQKMAEGLAFSISEYILPNLISFPVNEIGLIYGCRDDLKKLEDTLSVIRDVLLDAEDMQEKSHF VKNRIKKLEEVLYDADDLLDYFATEGLRRKLDTRGRIARQVCDFFSPSSNQLAFRHKIGHAIREIRGRLDEIANDISKFNFVREKAIVVGNRLEIR TRERERETDSFVMTSEVIGRDKEKKEIIDMLMGSGNSHHVSVVPIVGLGGLGKTALAKLVYNDESVVKQFEPRIWVCVSEDFDVKLIIQSILRCIT NEKVDDLEMEQLQTHLRKNLDGKKYLLVLDDVWNEDHRGWLQLERLLMVEANSSKIVVTARSSRVASAMGINEPYILNGLTEDQSWKLFESLAFGK GLDKVDSKLVKIGKEIINKCKGVPLAIKTLGSTMRSKVGESEWLSIQNNEIWSLSLEDSGILPVLKLSYDHLPTHLKFCFAYCSIFPKDYKIKYHV LIQLWMAQGYIQSSSQRQNLEYIANHYFRDLLSRSFFQEVEVDDFGNITNFKMHDFIHDLAKEVAGNHCCSITNANDENNYERVRHVSSQVERWRI PRLLLEEKGMRTLFISSLDSNRWRQNLIGSRKVIAKFRRIRALDLHGCAISKVQGSIGLLKHLRYLDLSGNYDMKHLPNSISNLVNLQTLILSNCW NLKELPRGVTKLINLRYLMINGCPKLRHMPLGLGKLTSLQMLSKFEVSLSRGCGRLHELSSLKDLRGEIEIKQLEKVRDATSVSKQAKLNEKPYLQ SLKLHWKKGKWKAVPRHITEEEHAELVLEGLQPHSNLKELRISNYGGAKLGSWMMGNVFLSSLPNLVSLTLINFERCQHLPELDQLSCLKFLHLQS LYAVENIG
50.	**>Cluster-12591.17748;orf1 len=359 frame:3 start:3 end:1079 1216948219|XP_021670348.1 putative disease resistance protein RGA1 [Hevea brasiliensis]** SLRTLSKFIVGDDKRSAKLGELNGLNNLRGKLLIENHENVRNSRSVSKEANLMGKPHLKSLTLNWGGDNFEEERGVVTDADKEDAEIFLEGLQPHP NLKKLKIEGYCGGEKPSWIMGSSLPNIVEISLRNCPNIRHLPPFHQLPCLKSVDIQNCPKVEYDDDGSSSSSSFSPSFPCLTHLTVIGCPKLTSMP CPPLLETLHLYYVSQKLLLCQLLTTTIPSSSPLSRLTDLSIWDMEGIESLPEGVPHNLTSLQKLEIRFCPRLLHLAVSGEIQHLTALENLTISNCE ELDLTHDGMNWQGLRSLRTIWITNIPKLVSLPEGLQHITTLQRVVIWYCPGLVELPEWTSNIPSFKIYNS*
51.	**>Cluster-12591.19940;orf1 len=192 frame:-2 start:577 end:2 1050614242|XP_017629897.1 PREDICTED: putative disease resistance protein RGA1 [Gossypium arboreum]** KILKLFRAKPHFSQDIEHPAKPHFSQEIVHLGRTCPRLLRLAEIQHLTALEDLRINNCEEVDLSDDGMNWQALSSLRKLHICNLPKLVSLPEGLQH LTALEHLIISEVDLSADGMNWQALSSLRRLTIAYFPKLVSLPEWLQHVTTLQTLDIYGCRGLVALPEWMSNLTSLNYLNIYGCPLLSERCQKDTGE
52.	**>Cluster-12591.21547;orf1 len=247 frame:-1 start:742 end:2 1216928687|XP_021677926.1 putative disease resistance protein RGA1 [Hevea brasiliensis]** VRISLHNFPSIRQLPPTLYQLPCLKYLDLGNLPKYNNGDGDGPSSSSSPSFPCLATLRISECPMLTSMPLAPLLETLNLHSVSEKLLCQQLSSSPL FRLRSLAIRHIEDIEALPLLRNLTSLNTLSINSCPRLLRLAEIQHLTALEDLQIFDCEEVDLSDDGMNWQALSSLRRLYIRDLRKLVSLPEGLQHV TTLQILEIWNCHGLVALPEWMSNLTSLKYLCISGCPLLSERCKKDTGEDWPNIAH
53.	**>Cluster-12591.19778;orf2 len=332 frame:-3 start:997 end:2 1344089145|XP_023928736.1 putative disease resistance protein RGA1 [Quercus suber]** SCEEVQGVPLALKTLGSLLKGKNKDSDWIAIRDSEFWKLKQGENDILPALRLSYEHLPSHLKLCFSYCSLFPKDFEFNNIMLVHFWMAQGLVRSSD EGQELEDICNQYINELCLRSFFQDVYVAGFIITFKMHDLVHDLALLIAKDEMSTINIPSQSISKTIRYMAFWDHVSCETVPSVLFNQTRLRSFKFW CTDDTYVVSNSFVDSCISRSKYMRSLDLGGVQFKVLANSIGDLKFLRLLNLSHNKSIKKLPKSIYKLQNLQTLQLHGCSNLEELPKDIGKYFINLR YFTFTTNQVSLEIGCLPTSLRFLHIFECENLKSLGEGLECLTAL
54.	**>Cluster-12591.17749;orf1 len=275 frame:3 start:3 end:827 1216929794|XP_021683312.1 putative disease resistance protein RGA1 [Hevea brasiliensis]**

	DIGDQYFEDLISRSFLQDVKVTSDNVYCKMHDLIHDLALEIAGPRYCSIAENSSVNISERVNHVSFGLLENVPTQLIQAKGLRTYFSTEYFWFLSS KLQARPNNLLLSKIRRLRVLDLHGCGFKKLPNLVGKMQHLRFLDLSWNIEMEVLPNSITNLVNLQMLNLSYCFGLKELPEGISKLISLRHLIIATC PKISRMPSGLGKLTSLRTLSDFVVDDDKRSAKLGELNGLNNLRGRLCIENLEKVRGSGSESKEANLMGKQYLKSLTLHWSWSR
55.	**>Cluster-12591.17643;orf1 len=103 frame:-2 start:433 end:125 747044432|XP_011089014.1 putative disease resistance protein RGA1 [Sesamum indicum]** PVSVPEIRFPSWIQHLTALEDLTIGHCKELDLTHDGMNWQGLRSLRRIVIERIPKLESLPEGLQHITTLQSVWIEDCPGLVDLPEWTSNIPSLFIP SLNIRR*
56.	**>Cluster-12591.20644;orf1 len=135 frame:-3 start:405 end:1 1000945246|XP_015581366.1 putative disease resistance protein RGA1 [Ricinus communis]** LPFLCILQACIPSCETSFIPQSNYSFLYPFSCSLLLIQVFGLLLCQNNMAEAIAYNVVADILFKMGDAIFREICSVYGMDDDLENLKETLITVNVA LLDADRRQEENHLIKDWIRKLNDVVYDADDVLDAFNTKN
57.	**>Cluster-12591.15362;orf1 len=205 frame:1 start:1 end:615 1344089145|XP_023928736.1 putative disease resistance protein RGA1 [Quercus suber]** NFFSSSSPLSFRHEMGRRMKALRERLDDISNEMSKFHYLTTRAEYAHVLHQREMTHSFLRPSDAIGRDNDKEHIKKYLLREGDGETVSIIPIVGIG GLGKTTLAKLVYNDDNVEKHFEMKIWVCVSEEFEVTKLAKDIIKNATGQNVSDYTMEQAQTCLRDTLRGKRFLLILDDVWNEDRRKWVELKNLLLD GVPGSKIIVTTRI
58.	**>Cluster-12591.10942;orf1 len=313 frame:-3 start:948 end:10 658005047|XP_008337657.1 putative disease resistance protein RGA1 [Malus domestica]** NIYGIESGAPIVNIGSKMSTLTHLTVWNIPELGLLLQNNKDLTSLEMLDCDQNLMISLDWVRNQVYLESLTIFSCPNLTSSMVSIQGLTSLKKLRI ESCDGMTSLPIGLGSCASLESLYVRRCANLVSVAEEDLRELKSLSSLTISGCKNLKRIRFERFLHCLPRLQSLRIGGFSDELDSFPFTINVDYIQQ LPQLQHLNLIGWPKIKSLPDQIQHFTTTLRTLYLFKFEELEVLPDWLGELSSLERLTILYCGNLKSLPSVTSIQRLSNLQSVYIDGSPILKERCLG DGPEAFKIPHFANTAYISIDGRQF*
59.	**>Cluster-12591.13749;orf1 len=283 frame:1 start:1 end:849 255556679|XP_002519373.1 putative disease resistance protein RGA1 [Ricinus communis]** KKLGKTSCFCRPVATGRLFLRNISRQHLHSQTNSTGLSSNISLAIFTLLLYQENMVEVIAFKVAANIVTNLGGAAFREICSVYGVVDDLNKLKEKL STIEAVLVDADRRQEKSHLIQLWIRRLNDVVYDADDLLDVFRTKNLQRQNEKANMQEVREFAFRYKLGRKIKKIRERFDEIAADMFKFNFVTERVI DRVRVDGRGRETDSHVDSIAIGREKEREEIINMLMCSSSEENVSIISIVGIGGLGKTTLTQLVYGDKRVVEHFESMVWVCVSEDFERKSLV
60.	**>Cluster-12591.11844;orf1 len=167 frame:-3 start:1067 end:567 1216928687|XP_021677926.1 putative disease resistance protein RGA1 [Hevea brasiliensis]** LSSSPLSRLRSLTIENIEDLGALPEDVLRNLTSLKTLYIWGCPRLLRLAEIQHLTSLEDLYISDCKELDLPEDGINWQALRSLRKLEIEKVPKLVS LPEGLQHVTTLQSIDITDCPGLVALPEWMSNLTSLKHLYISECPLLSERCQKDTGEDWPNIAHIPNIEID*
61.	**>Cluster-12591.13460;orf1 len=248 frame:1 start:37 end:780 1217040497|XP_021660275.1 putative disease resistance protein RGA1 [Hevea brasiliensis]** EFPFILKATTSLSSNISLAIFTLLVCQENMAEAILFNVFADLVTKLGVAACREVRSVLKDDLIKLQETLSTIDAVLVDADKRQEESVQIQLWIRRL NDVVYDADDLLDAFHTKNLQRENGRTKMQEVREFFSSSNQLAFRYKLGRKIKKIRERLVEIAKDMSMFKPLVTEVVIDQARVKDRDRETESCVEPI VIGRETEKEEIINMLMCSSGEENISIIPIVGIGGLGKTTLTQLVYCDKRVVKHFES

62.	**>Cluster-12591.10151;orf1 len=153 frame:1 start:1 end:459 1344017914|XP_023891722.1 putative disease resistance protein RGA1 [Quercus suber]**<br>LQTLRLVGCSNLEELPKDIGKYFINLMYFTFTTKHVSLEIGCLPTSLRYLGISACENLKSLGEGLECLTALRTLIIINCKSLERLPRGLSCSTTIQ<br>NLVLFGCPEIDTSELEEDTENLRLRLQFLAIGNLPKFEVFSRWIQGSADSLEYLGIG
63.	**>Cluster-12591.12699;orf1 len=231 frame:-2 start:695 end:3 1552276124|RVX18138.1 putative disease resistance protein RGA1 [Vitis vinifera]**<br>ERERERDDISNEMSKFHYLTTRAEYAHVLPQREMTHSFLRPSDAMGRDNDKKCIIKQLLREGDGETVSIIPIVGIGGLGKTTLAKLVYNDDVVQSR<br>FDLKIWVCVSEEFKVIKLAKEIVKIGSGEDVGDCTMEQAQTCLRDTLRGKRFLLVLDDVWNEDMRKWVELKNLLLDGVSGSKIIVTTRIPSVASIM<br>GSVPSHNLDGLPHEDCLSLFVKWAFNDGQEEQYPNLMKI
64.	**>Cluster-12591.20650;orf1 len=158 frame:-3 start:476 end:3 1217040497|XP_021660275.1 putative disease resistance protein RGA1 [Hevea brasiliensis]**<br>SFIHHFPYYLLLPFNSEFLLCQNNMAEAIAFNVVADLVTKLGVAALHEICFVYGLKDDLKKLQETLSTINAVLLDADRRQKNNNQIQLWIKRLNDV<br>VYDADDLLDEFHTKNLQRENGRTKMQEVREFFSSSSQLAFRYKLCGKIKKIRERLDEIVANM
65.	**>Cluster-12591.14369;orf1 len=245 frame:-1 start:735 end:1 1216928685|XP_021677917.1 putative disease resistance protein RGA1 [Hevea brasiliensis]**<br>QLPCLESLYLDNLPIVEYGGDGDGDGDGDGSSSSFPCLTSLEIRDCPMLTSVPLAPFLEELILETVSEKLVCQQLSSSPLSRLSYLVITNIEDLEA<br>LPEDDVLRNLTSLKTLEIWGCPRLLRLAGIQHLTALEGLTIYGCKEFDLSDDVMNWQGLRSLRKLQIGGFRKLVSFPEGLQHVTTLQSIDISHCPE<br>LVALPEWMSNLTSLKDLRIFGCPLLSERCKKDTGEDWPNIAHIPTIDIDFKSV
66.	**>Cluster-12591.9835;orf1 len=261 frame:3 start:3 end:785 1220043759|XP_021807738.1 putative disease resistance protein RGA1 [Prunus avium]**<br>PPTLQSLTIKHFLGCNFPLWMMISNLSYSGNPQLPQSLVSVELIGCSNCEQIPTLGELQFLQFLEIDGMANVKYIGKEFYSHSSGGDGSTKSCTLF<br>PKLKKLSFREMHNLVEWSEAAATTSTASSERGGAVVVFPCLEELTINNCERLQTAPSHFPSLLKLNIYGIESGAPIVNIGSKMSTLTHLEVAHIPE<br>LGLLLQNNKDLTSLTIACCPILTSSMVSIQGLTSLKSLSIYSCYGMTSLPIGLGSCASLEILVVYGCAN
67.	**>Cluster-12591.16752;orf1 len=553 frame:2 start:2 end:1660 1585749012|XP_028070224.1 disease resistance protein RGA2-like [Camellia sinensis]**<br>EQLNALIRLRHLYFDSANMSMMPISMGRLICLQTLPCFVVGKYMGRRIEELGSLINLRGKLDIRNLERVSGKEEAQKANLTKKTKVYKLELSWSSN<br>EREGNINDENVLEGLQPPPNLNSLTIKEFRGSKFPSWLMIANTSYNGNSFLPLSLGEISLMNCTKCERMPTLGNLPFLKSLEIRGMLNLKCIGTEF<br>YSGYRSGDGDRRLAFFPSLQKLALENMPNLVEWKLVATTTEGAAVVFPCLEELSICECEQLQIVPCHFPSLRKLKIDGIKSGKPIVDICSKMTSTL<br>THLVIENVRELGFLPNGLCTLVALETLRILYCPNLKSIPSIQGLTSLKNMWIDGCHGLTSLPIGLGSCTSLEKLSLFNCGNLVSIAEEEEDLSELS<br>CSLGGLRIIKCPKLKSWPLDKFPHRLPKLEELGIGGFGEELDSLPLNLNLNIDSFPQLEDLRLYGWPKLKYLPDQLQHLTTLRELALVSFEGLEVL<br>PDWLGNLSSLHTLSLWFCKNLKYLPSVESMQRLTNLTSLCVKYCPLLEERCNKGSGPEWSKISHIQLIIINDR
68.	**>Cluster-12591.17639;orf2 len=397 frame:1 start:409 end:1599 1389659480|XP_024929347.1 disease resistance protein RGA2-like [Ziziphus jujuba]**<br>EIESSDGKSEATNPTVNNRGNKAATIPSSSPLSRLTHLSISFMEDIESLPEGVPENLTSLKKLEIVWCPRLLHLAVSGEIQHLTALEDLQISNCKE<br>LDLTDDGMNWQGLRSLCNMEIEGIPKLVRLPEGLQHITTLKSVDILNCPGLVEFPPFHQLPCLKTLTLRELPKVEFDDGSSLSSSSFSPSFPCLTH<br>LDIYDCPKLTSMPCLPLLETLKLERVSHKLLCQLLTTTTTAATSSPLSKLRSISIRGMEDSESLPFFPKGVPQNLISLEKLEIQRCPRLLHLAVSG

	EIQHLTALEDLTIWNCEELDLTHDGMNWQGLRSLREIVIWGIPKLVSLPEGLQHITTLQSVDIRDCPGLVDLPPFYQLPCLSHLKISNCRKLTSMP<br>CPPQLARPQEPL*
69.	**>Cluster-12591.24175;orf1 len=745 frame:-2 start:2237 end:3 1585792596|XP_028090744.1 disease resistance protein RGA2-like [Camellia sinensis]**<br>LCQEATSTFSLFSSLRDLICLRKFASTFSFPMAEIILSAITEGVVGKLTSLASQEIGLAWGVKSELNKLNNTVSSINAVLLDAERQQTKSLAVKDW<br>LEKLKDVVYDIDDVLDDFQTEALQKTVKTRWGMLKEVRNFFSKSNPFAYRLKMGHMMKGIRERLDAIESNRKFQLSEHVGETQIQTRETHSFVQES<br>EVIGRDKDKHNIVELIMTNNKQANLCVIPIVGLGGLGKTTLAKLVYNDKDIAQSYDEYRLWVCVSEDSKLSRLIEKIIKAATRESCEGLDLEQLQD<br>KIRGILYAKKFFLVLDDIWNDDPKWWADLRELLVGGAVGSTIIVTTRSLKIGSIVGTIESYRLGDLLLEDCSSLFFKFAFKEGKAREHPELMKIGK<br>EIVQKCGGVPLAVKTLGSLLYMKTDVGEWSYIKNNDIWVLEQGENDILPALRLSYERLPSYLKQCFAYCSIFPKDYDITKDELIYLWMAQGLLQSR<br>DKNQRLEDIGNRYFNELLSRSFFQDVERNFDNSVWTFKMHDLVHDLARSVVGKEFFDADIDSSAIPENVRHLIFDGDDLSNKEFPKSLVFAKKVRS<br>FAFSLSAGPISQNFVDTLIMSFMCLRVLELSCSTFEVLPSFISKLKHLRYLRLAGNDRLKALPNSICMLLNLLVLDLYGCDQLQDLPRDIGRLTSL<br>RCLAITSQGVSMPESGLRCLTSLQNLWIFNCNRLESLPNGIQHLTALRELRITECSRLTSLSNGMKGLAALES
70.	**>Cluster-12591.19118;orf1 len=286 frame:2 start:2 end:859 1130654543|XP_019704715.1 disease resistance protein RGA2-like, partial [Elaeis guineensis]**<br>LDLRQCGIEKLPNLVGKMQHLRFLDLSWNSKMEVLPNSITNLVNLQTLNLSSCGGLKELPEGISKLISLRHLKIIGCHKITCMPRGLEKLTSLQTL<br>NLSSCFGLKELPEGISKLISLRHLIIADCTNINRMPRGLGKLTSLQTLNLSSCFGLKELPEGISKLISLKHLIIADCTNINRMPRGLGKLTSLQTL<br>SKLIVGDDKRSAKLGELNGLNNLKGDLRIENLKNVRNSGEESKEANLMGKPHLKSLTLNWGYYFGEADKKNAEILLEGLQPHPNIKELKIDGYC
71.	**>Cluster-10015.0;orf1 len=886 frame:-1 start:2849 end:192 1217046002|XP_021663086.1 putative disease resistance protein RGA3 [Hevea brasiliensis]**<br>NSFFILLHQNHHFKIPNSEKMAETFLFSIAEKILEKLYSDTFQQIGLAYGVKRELKKLEHILFTIKAVLLDAEEQQTKNRQMSIWLESLRVACYDA<br>DDVLDEFTHRVLRQKAASTRGGSLISKVTNFFSLSDIKFRLVMGRKIKIIRERLDDIAANKAQFHLTERNVDHRHVVTGEREMTHSFVRPSDVIGR<br>ETEREEIVQQLLTLSSGRGIDRNFSVVSIVGIAGLGKTTIAKLVYNDERVNMCFDLKMWVCVSDEFNLVRLTKEIIKSATSENYSELSIDHLQTRL<br>RDILQGQRFLLVLDDVWNDDVEKWNKLRELLLDGATGSKIMVTTRSHSVASVMGTVPPYNLKGLSEQACWSLFMNCAFKDREDRQHANLESIGQEI<br>VKKCRGVPLAVRSLGSVLFMKTNERDWLFIRDNDIWKLEQRENDILPALKLSYKQLPSYLKECFAFCSIFPKDYKFDSIELVEMWIAQSFIYSSNE<br>NEELEDIGNRYIKELWSRSFFQDVEEHDSLILFKMHDLVHDLALLVAHADYGAVNFPRRNVSNSVRHVLFRDRNLSLEEVSTFLHDQNSLRTISFP<br>LDGVGSPGKPFVETCIKRSKYLRVLDLSNSTFEVLPSSVGSLKHLRYLDLSGNVTIKKLPNSICKLLNLQTLRLSGCLELNELPKDIGNLTCLRCL<br>YVTTKQTFLELDKGIGCLTSLRVLALAECSNLKSLGEGMQCLTALRTLSFLSCENLESLPSSMKYLPALESLIIANCKKLNLMEWEKDDDIHDIKL<br>MSFRSLYINKLPQFEALPKWLQGARNTLQYICIGNCDNFMVLPEWLQNLTSLQKLVIMKCPRLSASLPEGMQRLVSLKELQIGGCPEMSRRCNPVT<br>GEDWYKISHIPEITLDWKKIR*
72.	**>Cluster-12591.18471;orf1 len=306 frame:1 start:442 end:1359 1217046002|XP_021663086.1 putative disease resistance protein RGA3 [Hevea brasiliensis]**<br>DHSSSSCYYPSPKLITFSQKHIKKMAETFLFSIAENILGKLASHTFQQIGLTWGVERDLEKLEDTLSTIKVVLLDAEDRQSKNRELSLWLERLEDV<br>CYDAEDVLDEFAYEALRKQVVARGSLKTKVHNFFSHSDLKFRFVMGRKIKEIRERLDEISANKAKFHLTVRDGYWPVVTREREMTHSFIREVIGRD<br>VERENIVELLTSRDIDKNFSVVPIVGMGGLGKTTVAKMVYDEERVKGCFELRMWVCVSDDFSLKTLTKAIIESATNSTLSDFSIDQLQYRLRHTLE<br>VATNCRVYRRTCGCLLH*
73.	**>Cluster-12591.17751;orf1 len=82 frame:-2 start:247 end:2 1050612991|XP_017629256.1 PREDICTED: putative disease resistance protein RGA3 [Gossypium arboreum]**

	WHELARPQEPPYYFHLGIVIENIPKLVSLPEWLQHITTLEFLTISDCKELDLTDDGMNWQGLRSLRKVEFRNIPKLVSLPEG
74.	**>Cluster-12591.21276;orf1 len=137 frame:-2 start:411 end:1 1173844450|XP_012086134.2 putative disease resistance protein RGA3 [Jatropha curcas]**<br>GMQRLTSLRNLGIVNCKSLERLPRGLNCSTTLENLVLQGLAEMEFEEDLPLSLRYLLIINLPKMEALPRWLQGSADSLERLVIYDCSQLSTLPSCLQDFTSLRTLKILKCPQLSALPEGMNHLTELTIVDCPELSAL
75.	**>Cluster-12591.17642;orf2 len=270 frame:3 start:3 end:812 1009170363|XP_015866159.1 putative disease resistance protein RGA3 isoform X1 [Ziziphus jujuba]**<br>LLNCPNIRQLPPFYQLPCLKTLTLKYLPEVEKDGSFSSFSSSFPCLTTIKWCRKLTSMPLAPLLETLELHSVSQKVLFQLLATATRTTTAATIPSSSPLSRLTDLSIRFMEDIESLLPEGMPQNLTSLKKLSIVCCPKLTSMPLAPLLEKLEISYCPKLTSMPGPPLLETLKLVGVSQRLLCQLLTTATRTTTAATIPSSSPLSRLTDLSILFVEDIESLPEGVPQNLTSLKNLRILQALDCCIWRSRERFSISLPLSISKSLNAKNLI*
76.	**>Cluster-12591.17641;orf1 len=99 frame:3 start:3 end:299 1343987837|XP_023875851.1 putative disease resistance protein RGA3 [Quercus suber]**<br>WQGLRSLRRIHIENIPKLVSLPEGLQHITTLQHVVIYNCPGLVDLPEWTRNIPKLLIPSLNIRFVQLSFLTSLFFSLQSQFLKLGHFLSYYSFIYLLQ*
77.	**>Cluster-12591.19088;orf1 len=199 frame:1 start:1 end:597 1216278241|XP_021623640.1 putative disease resistance protein RGA3 [Manihot esculenta]**<br>FHDVDLSCQNVPNILLNQNRLRTILFPYAGVSVTSNSFVETCISRFKYMRALDLCCSSVEVLSSSIGNFKHLRHLNLYNNKSIKKLPKSICKLQNLQTLYLHGCSELEELPKDIGKYLISLRVLFVTTKQTSLEVGCLPTSLRHLEFSDCENLKSLGEGMQRLTSLRTLGITNCKSLERLPRGLNCSTTLENLVLSELA
78.	**>Cluster-12591.19086;orf1 len=202 frame:2 start:2 end:607 1216278241|XP_021623640.1 putative disease resistance protein RGA3 [Manihot esculenta]**<br>TFLELGCLPTSLRILQFVDCENLKSLGEGMLRLTALRTLRILNCNSLERLPRGLNCSTTLENLLLSRLAEIDTMEFEEDEGDLSLSLRFLGFYKLPKMEALPQWLQGSADSLEWLLIGDCSKLSTLPLWLKDFTSLRTLIIGECPQLSALPEGMNHLTELKIVDCPELTNRCQPETGEDWPKIAHVPKITLDDKIVKSPEN*
79.	**>Cluster-12591.7016;orf1 len=110 frame:-2 start:331 end:2 1216967079|XP_021680183.1 putative disease resistance protein RGA3 isoform X1 [Hevea brasiliensis]**<br>KCEEVDLSDDGMNWQALSSLRDLHIQNVPKLVSLPERLQRVTTLESIYITGCHGLVALPEWMGNLTSLISLFIMGCHLLCERCKKDTGEDWPNIAHIPNITIDEESVDDP
80.	**>Cluster-12591.24083;orf1 len=132 frame:1 start:1 end:396 1365976378|XP_024187075.1 putative disease resistance protein RGA3 [Rosa chinensis]**<br>SFPLDINIRQLPQIEYLALCGWPKLKSLPHQLQHFTTLKQLYISDFEGLEVLPDWLGNLSSLVYLSIGGCRNLKYLPSVESMKRLTNLRTFRVEDCPLLKERCNKEEGSGGPEWSKISHIPDIRINGWKMQ*
81.	**>Cluster-12591.13674;orf1 len=149 frame:2 start:2 end:448 1343983468|XP_023873545.1 putative disease resistance protein RGA3 [Quercus suber]**<br>MNWQGLRSLREIKFVNIPKLVSLPEGLQHITTLQSVEIIDCPGLVDLPPFYQLPCLTHLAIKWCPKLTSMPCPPLLEKLDLEGVSQKLLRQLLTTTIPSSSPLSRLTDLSIEKMEGSESLTFFPEGVPQNLTSLKNLEIRDCPRLLHLA

82.	**>Cluster-12591.19111;orf1 len=232 frame:3 start:3 end:698 1083912822|XP_018622398.1 PREDICTED: putative disease resistance protein RGA3 [Nicotiana tomentosiformis]** LVHDLALLITKDEFLSMSTRTQSIPKMIRHVSFYDNDLSCKEVPSVLLNQNRLRTILFPDKNVSVPSNSFVETCILRFKYMRTLDLEGSSFEVLHDSIENVKHLRLLSLAHNKSIKKLPKSIYKLHKLQTLHLAGCSELEELPKDIGKYLISLRHLCVTTKQTSLEVGCLPTSLRSLEIGECVNLKSLGEEGMQRLTALRTLAIVECKSLERLPRGLNCSTTLENLVLSELA
83.	**>Cluster-12591.19112;orf1 len=168 frame:-2 start:505 end:2 1173844450|XP_012086134.2 putative disease resistance protein RGA3 [Jatropha curcas]** RTRGTAQRFWENLKCLGEGMQRLTSLRTLWIVNCKSLERLPRGLNCSTTLENLVLQGLAEIDLMEFEEDKEGNLPMSIRSLGIGNLPKMEALPRSLQGSADSLEWLWIYDCSELSTLPSWLQDFTSLRTLEIGRCPQLSALPEGMNHLTELKIEDFPALTGEDLLKIA
84.	**>Cluster-12591.14663;orf1 len=333 frame:1 start:1 end:999 1585792616|XP_028090754.1 putative disease resistance protein RGA3 [Camellia sinensis]** QNIVQLIMDNNNQTDLSVIPIVGLGGLGKTTLAKLAYNETEISESFELKSWVCVSEDSKLNKVIERIIESATGEKCEGLNLEPLQNKLRDILFSNKFLLVLDDLWEDDPRWWTELRGLLVGGDVGSTVIVTTRSLKIGSIVCPTTASYILSELSFDDCLSLFLKCAFLEGKEHPELMKIGKEIVKKCGGVPLAVKFLGSLLYMKTDVGEWSYIMNNDILAWKQKENDILPALRLSYEQLPTYLKPCFVYCCIFPKSAEIQRFVLINFWIAHGLLQSHDKNERLEDIGNRYLNELLSRSFFQDAKRFFDNAITSVKMHDLVHDLARSVAGTESL
85.	**>Cluster-12591.21693;orf1 len=183 frame:3 start:3 end:551 1585686519|XP_028117281.1 putative disease resistance protein RGA4 [Camellia sinensis]** GAANETPYLVGIAKKMSKRCGGVPLALTTLGAMMYSIEANKWPSILESFSWDIEKGIMPALQLSFNNLPSPCLKQCFAYCSIFPKDSILEKDDLIQLWITQGFIPTSEGSNTLMEDIGNDYFKTLLWNSFFQDVEMDKYGNIKTCKMHDLMHDLAQTLSKSECLILDANDPNVSIEIRHLSLA
86.	**>Cluster-12591.21483;orf1 len=297 frame:2 start:2 end:892 1585686519|XP_028117281.1 putative disease resistance protein RGA4 [Camellia sinensis]** LCVISVVGLGGLGKTTLAQLVYKDPSVTSHFSQLMWICVSNDFDVTRILKEMLERLGIYVGEASNIEGLIQVLKKQLDGKKYLLVLDDVWNTDEAKWESLRNSLLGIGAAKESKILVTTRNVEVASTMTKTLLTHHLSVLLDEDSWSLFHQRAFATGGAANETPYLEGIAKKMSKRCGGVPLALTTLGAMMYSIEANKWPSILESFSWDAKKGIMPALQLSFNNLPSPNLKQCFVYCSIFPKDFIFEIDDLIQLWIAQGFIPTSEGSNTLMEDIGNNYFKTLLWNSFFQDAEMDKYG
87.	**>Cluster-13244.0;orf1 len=131 frame:-2 start:394 end:2 1552146455|RVW16612.1 putative disease resistance protein RGA4 [Vitis vinifera]** LSSIPFASSSSFHTHLSFHPFHLLLNLNMAEAILYHMIATLLGNTTSFAMKEIALAWHVESDLEKLNNLSSIHAVLQDAEDKRVKNHLLNDWLRKLKNFVYDAEDLIDEFQYEGLRRPVLKSESIGRKSTR
88.	**>Cluster-12591.14642;orf1 len=296 frame:3 start:351 end:1238 1079229790|XP_018505451.1 PREDICTED: putative disease resistance protein RGA4 isoform X1 [Pyrus x bretschneideri]** GRRSGHHALIMGNMVISLTNIVQISLNKFPSIRHLPPFYQLPCLKSLYLRDLPKVEYGSDGSSSSSSSFFSPSFPSLTSLSISDCPMLTSMPLHPLLERLSLESVSEKLLCQLFTTTTTTIPSSSSSSSPLSRLSTLKIEGMEDFEALPEDVLRNLTSLKTLYIWHCPRLLRLAEIQHLTALDALYIRGCPSLLRLAEIQHLTSLKTLGICGCPRLLHLAEIQHLTALEVLYIEDCEEVDLTDGGLNWQGLSSLRDLEIEKVPKLVSLPEGLQHVTTLQRIEFDDCPGLATLPEWM
89.	**>Cluster-12591.14631;orf1 len=92 frame:-2 start:1277 end:1002 802600076|XP_012072751.1 putative disease resistance protein RGA4 [Jatropha curcas]**

	VPKLVSLPEGLQHVNTLQSIEIYDCPGLVALPEWMRNLTSLKVLWIDGSPLLWERCQKDTGEDWPNITHIPIIDINWKSVQMREGTDDIVV*
90.	**>Cluster-12591.17746;orf1 len=76 frame:-1 start:377 end:150 1173824992|XP_012072753.2 LOW QUALITY PROTEIN: putative disease resistance protein RGA4 [Jatropha curcas]**<br>NWQGLRSLRTIWISNIPKLVSLPEGLQHITTLESVVIEDCPGLVDLPEWTSNIPDFEINSGQATSLTSRFGIVLV*
91.	**>Cluster-12591.13479;orf1 len=72 frame:1 start:1 end:216 1173824992|XP_012072753.2 LOW QUALITY PROTEIN: putative disease resistance protein RGA4 [Jatropha curcas]**<br>LQHVTTLQSIHITDCPGLVTLPEWMSNLTSLKHLVINGCPLLSERCQKDTGEDWLNIAYIPNIRIDLQQIQ*
92.	**>Cluster-12591.18177;orf1 len=166 frame:1 start:1 end:498 1445363470|RDX62544.1 TMV resistance protein N, partial [Mucuna pruriens]**<br>KEIEGIRLEAFKFDERFQPEAFMKMKNLRFLFISVPFVVKQFPEGISNILDELKILEWMRYPREHLALDFPHHKLVHLSLINSAIKLLWNGKKVLQ<br>YLKSIDLSLSEDLRKFPDVTGLPNLERLSLRSCRQLVKIHPSVWVHKGLKFLDLHGCRNLKSFPNKIEME
93.	**>Cluster-12591.13148;orf1 len=109 frame:-3 start:329 end:3 1343992116|XP_023878073.1 TMV resistance protein N-like [Quercus suber]**<br>VFINLSMASSSKLDLFEYMPTQKIIKTASTSLPFSSSSSASAPNLKWTYDVFLSFRGEDTRNNFVDHLFMKLTEKGINTFRDDEKLKKGKEISPEL<br>LKAIEGSRFAIII
94.	**>Cluster-12591.12001;orf1 len=881 frame:2 start:899 end:3541 1371540299|PSS36205.1 TMV resistance protein like [Actinidia chinensis var. chinensis]**<br>CTMGRPEADLIKDIVEDVLDHLNRRTDLVVAKHPVGFETRVRALDSKMLVHNLGRVQIIGFCGMGGLGKTSLAKAVFNQFFLAFKGKSFLANIREI<br>SKQPDGLLRLQKQLLSDILRGKMENISSVDRGINVIKERLCNKRVLVVLDDVDHLDQLHALGVKLTSAQAREGDWGLGSRIIITTRDRHLLENIEV<br>DSLHDVSELNDDESLELFSRHAFKRSRPEEDYDELSKDVVSYCKGLPLALEVLGSHLSKRSIHVWTSALEKLKRIPHGDIQKKLRISFDALSDETE<br>KDMFLDIACFFIGMDKDDVIKILDGCEFYAENGITVLLSRCLMRVNKYNKLVMHDLLRDMGRGIVREESPKNPGKRSRLWFCDDVLDVLRNQTGTD<br>DVEGLVFKRPTTNELCAEAFAKMQGLRLLQVSNVHLNGDYGNISKKLRWLFWHGFPLKFIPNGVHLENLIAIDMRNSKMIQLWEGIKALERLKVLK<br>LSHSHNLLRTPDFSRVPNLEKLVLKDCTSLVEVDPSIGCLNQLHMLNLRDCKSLEVLPNSICRLRSLEILNLSGCSKFEKLPDDLGMVESLTELLM<br>DGTLIKEVIQVPRSIVRLKNLKKFQKCGFQGSPSSFRDSLFLSWESPRKSLALIKRESLGLSPGEHPFGVSTISYSSSKELTLYNCKSPNEDTMKG<br>ISLSSLVSMGLGSNNVFSIPASSSSLPNLNSLQLDHYMRFQSMPDLLEGLMIENAGHCTPVVSSKGQHQLNQAIDYLHQSYTKPLIDKQSQQLTIE<br>ELEVLRYKLPELVFKQPVPSAPPASVLLSTSARSFHSTSTSTNDTWRPICSSSPGPVCADDSSLEDRTTAMKIARGIILPRDDEFLANLYDHVLSV<br>CSPSSIILFLFFSFSR*
95.	**>Cluster-12591.13890;orf1 len=242 frame:-1 start:727 end:2 645235447|XP_008224273.1 PREDICTED: TMV resistance protein N-like [Prunus mume]**<br>LGGIGKTTIAKVIYNQISYQFQGCSYLANVREKYEKEGNLVPLQKQLLYEILKVQDFQIFDDDNGSSVIRNRLKNQKVLVILDDVDHVNQLEKLAR<br>NGEWFGRGSRIIITTRDQHLLSKNEGVQRYEVRECDSDKSLEIFSWKAFKKNHPPEDYMKLSNDVVRYTKGLPLALTVLGSHLCGRSKDEWRGAIS<br>QLSNVPNREINDILKISYEGLEELQKDVFLDIACFFIGKKEDEVMEKLDS
96.	**>Cluster-12591.8317;orf1 len=182 frame:-3 start:831 end:286 1344086353|XP_023927284.1 TMV resistance protein N-like [Quercus suber]**<br>FNIQVLIDKSLITVSAKNELMMHDLVRDMGRYIVHQKSPTNPGKRSRLWNYEDICRVLEKNMGTEEIECIKLTTCGFNSSLNAEVFTKMKNLRFLV<br>ISTQFRLYSFGMPIPKGLDNFPDELKILIWLGYPYEHLPLDFSHNKLVHLSLILSNIKLFWNGRKVRARVTCLLFLSCFTCLLFP*

97.	**>Cluster-12591.11179;orf1 len=103 frame:-2 start:311 end:3 1104673975|XP_019071901.1 PREDICTED: TMV resistance protein N-like [Vitis vinifera]** SYNPFCSKLGSFPNKIQMESLETLYLFYCKKMEEFPKVHGNVKRLSGLMLASTEIKELSSSIVNFTALKILGLFSCFNLVCLPSSICKLKYLELLNLSGCSNL
98.	**>Cluster-12591.18982;orf1 len=309 frame:3 start:3 end:929 1104676183|XP_019072219.1 PREDICTED: TMV resistance protein N [Vitis vinifera]** LPSSSASPPNLKWTYDVFLSFRGEDTRNNFVDHLFMNLTQKGINTFRDDEKLERGKEISGDLLKAIEESRFAIIIFSENYASSSWCLDELVKIIDCTKETGQIAYPVFYNVDPSNVRKREGSFAKAFDKHDKTNVASEKMEKWRKALSHAGSIAGWDTRGGHEAKIIQEIVEKIFDKLSHALLHVDDHLVGMKIRVEEINTRLNLGSNDVKFVGIHGLGGIGKTTTAKVIYNQISYQFEGCSYLANVRETYEKEGNLVSLQKQLIRDILKVEVDIGNDDRGSSIIQHRLNNQKVLIILDDVDHVNQLEK
99.	**>Cluster-12591.27284;orf2 len=53 frame:3 start:3144 end:3302 1552255903|RVX01170.1 TMV resistance protein N [Vitis vinifera]** DKQNRLNNKKVLIILDDVDHIKQLLKLATNGKWFGSGSRIIITTRDRHLLNS*
100.	**>Cluster-12591.12282;orf1 len=246 frame:-3 start:739 end:2 1343992116|XP_023878073.1 TMV resistance protein N-like [Quercus suber]** ERDLIRKKRREERFDIKRERERREVEDLPTQKRINLSMASSSRLDLFDQVIDLFEQGKEVQALQVIDLVEQGKEVEALQVIDLIDQGKEVEALQVIDLIEDLPTPTSLPSSSSSSASPPNLKWTYDVFLSFRGEDTPNNFVDHLFMKLTEKGIHTFRDEERLERGKEIYGDLLKAIEESSTLDLFNYMPIQEIITTASTSLPSSSSSSSSIDPYQDLSDGDYLWTDEDLSRPRLSKSITTASTSLP
101.	**>Cluster-12591.17829;orf1 len=241 frame:-2 start:723 end:1 1217012279|XP_021645648.1 TMV resistance protein N-like, partial [Hevea brasiliensis]** EDYTKLSNEVVNYAKGLPLALTVLGSHLCGRSKDAWRGAIEELKIIPNEEITGILEISYKGLNKTQKDVFLDIACFFIGEDKDEVMKKLHSFDFFPSINIQVLIDKSLITVSAENELMMHDLVRDMGRYIVHQESPKNLGKRSRLWNTEDIRRVLENNMATEAIEGIRLNAFMSGWRFKAEAFMEMKNLRFLECYGYPCEHLALDFPHDKLVYLSLDHSSLKLLWNGRKADLPHLKSIDLS
102.	**>Cluster-12591.17821;orf1 len=126 frame:-3 start:547 end:170 1025260410|XP_016490974.1 PREDICTED: TMV resistance protein N-like [Nicotiana tabacum]** LNLPSFNGLPSSLTVLNLSWCDLTDETFPSNIECLSSLETLVLSGNNFVSLPANITQLSLLTKLDLSSCRNLEKLPESLGDLESLVDLDASETAIKEVPASIQFLDKLRATFFLWFTVLIDRSGS*
103.	**>Cluster-12591.17827;orf1 len=76 frame:3 start:921 end:1148 1570361011|XP_027928396.1 TMV resistance protein N-like isoform X1 [Vigna unguiculata]** NSFSDSFSERWGSIEFLWNGRLALLPRLKSIDLTNSERLREFPDVTGLPNLERLSLRGSSIKSLWNGRQADLPRI*
104.	**>Cluster-12591.17838;orf1 len=72 frame:3 start:3 end:218 1343965818|XP_023909153.1 TMV resistance protein N-like [Quercus suber]** EILHSFGFFPSSSIPVLIEKSLVTVSAKNELMMHDLVRDMGRHIVHQESPKNPGKRSRILRTLGKYWKRIW*
105.	**>Cluster-12591.23536;orf1 len=84 frame:2 start:2 end:253 1103927420|XP_019059595.1 PREDICTED: TMV resistance protein N-like [Tarenaya hassleriana]** SLETLDLSGNNFESLPTNISQLPLLTKLDLIGCERLKLVPELPSRISIYTPGCTYLNGPHGEVFFRALRLIYDALDVEVAAAT*

106.	**>Cluster-12591.17815;orf1 len=117 frame:-3 start:351 end:1 1344017306|XP_023891406.1 TMV resistance protein N-like [Quercus suber]**<br>IEMESLETLYLHACFEMKEFPEVHGNMKRLSKLVLSETAIKELPSSIENFTPLKILRLYGCKNLVCLPSSICKLKYLEQLHLTDCSALEKLPENIGDLESLVELRASFTSIKEVPSS
107.	**>Cluster-12591.14206;orf1 len=59 frame:1 start:43 end:219 1104509053|XP_019073754.1 PREDICTED: TMV resistance protein N-like [Vitis vinifera]**<br>EIVFPIFYNMDPSNARNQKERFAKAFDEHEKKNSEKMKKWSEALSQAGKLFGWDCRDR*
108.	**>Cluster-12591.17822;orf1 len=179 frame:-3 start:537 end:1 1344079141|XP_023923534.1 TMV resistance protein N-like, partial [Quercus suber]**<br>FPKVHGNMKRLSKLVLSYTEIKELPSSIENSTSLKILELKNCKNLVCLPSSICKLKYLEELNLNYCSALEKLPENIGDLESLVELDASGTTIKEVPSSIQSLVKLRILSFGWCRGLKLPSFNGLPSSLTDLNLSCCNLTDETFPSNIECLSSLKTLDLSGNNFESLPTNITQLPLLTRL
109.	**>Cluster-12591.32346;orf1 len=146 frame:3 start:105 end:542 1344041313|XP_023908098.1 TMV resistance protein N-like [Quercus suber]**<br>NKKGKKKCEIAKWAGLSSSFPYLLLPRASPQKLNFDVFLSFRGEDTRKNFVDQKGINTFRDDENLERGKQISHDLLKAIEQSRFAIIIFSENYASSTLCLDELVRSLIAPNGGKRYHGQALFLSGIHGRDYWTLQSIFLNQTRLP*
110.	**>Cluster-12591.24230;orf1 len=92 frame:2 start:2 end:277 971574723|XP_015170539.1 PREDICTED: TMV resistance protein N-like [Solanum tuberosum]**<br>SRFAIIIFSKDYASSSWCLDELVKIIDCTKETGQIVYPVFYNVDPSNVRNQKDSFAKAFDKHKKKNLALEKMEKWKDALSKAGRLSGWDTR*
111.	**>Cluster-12591.18646;orf1 len=723 frame:3 start:507 end:2675 731374260|XP_010652777.1 PREDICTED: TMV resistance protein N isoform X2 [Vitis vinifera]**<br>LSINLYLFAVQMAEKLLVLNLHDCRNLTTIPDLSGHLVLEKLILENCEGLIKIYKSVGDLSTLLLLNLKGCSNLVEFPTDVSGLKHLKKLILSGCRNLTELPEDIHSMKSLRELLVDETAIVKLPDTIFHLQKLEKFSLSACRSLKELPDCIGKLSLLRELYLNDSALEKIPDSIGSLVRLEKLSLARCSSLTSIPDSLGSLKSLIELFLNGTSIIEIPSSIRSLTHLKTLTISHCRSLSKLPDSIEGLASLENFWLEGTLIKEVPEQLGSLTMVKQLEMRNCKLLGSIPKSIGKMLSLTSLALTNSAITELPESIGKLERLVTLQLNNCKQLSRLPNSIGNLNSLRYFYMEDTAVTELPQDFGMLSSLQILKMMKSPHSEEHLKISEHTVLDNPTAQESPKLVVLPTSFSNLSSLKELYARGWRISGKIGDDFEKLSTLEFLDLGKNSICSLPSSLRGLSNLKKLLLPHCKELKSLPPLPSSLVKLDVADCTALESISDLSDLKSLQELNLTNCKKIIDIPGLECLRSLSRLYTSGCNACFLAVKRRLSKVALKKLRYLCVPGSEIPSWFVQEIPRFTTIKNRNIKGVVIGVVISLDQQKHDNFRNKLPAIVDVQAKIVRFNDTIFTTVLYLLGVPETDDDQLYLCQFKEYNQLVFMLRDGDSLHIAIRDKPLFNGLSLKKYGIHLIYENDDDIVDDDDEEWLHESKQSVSYRLAKFFNSL*
