# Supplementary figures and images for: Analysis of R Genes Related to Blackcurrant Reversion Virus Resistance in the Comparative Transcriptome of Ribes nigrum cv. Aldoniai
Source: Plants (Basel). 2022 Nov 16;11(22):3137. doi: 10.3390/plants11223137 (PMC9692259; doi:10.3390/plants11223137)

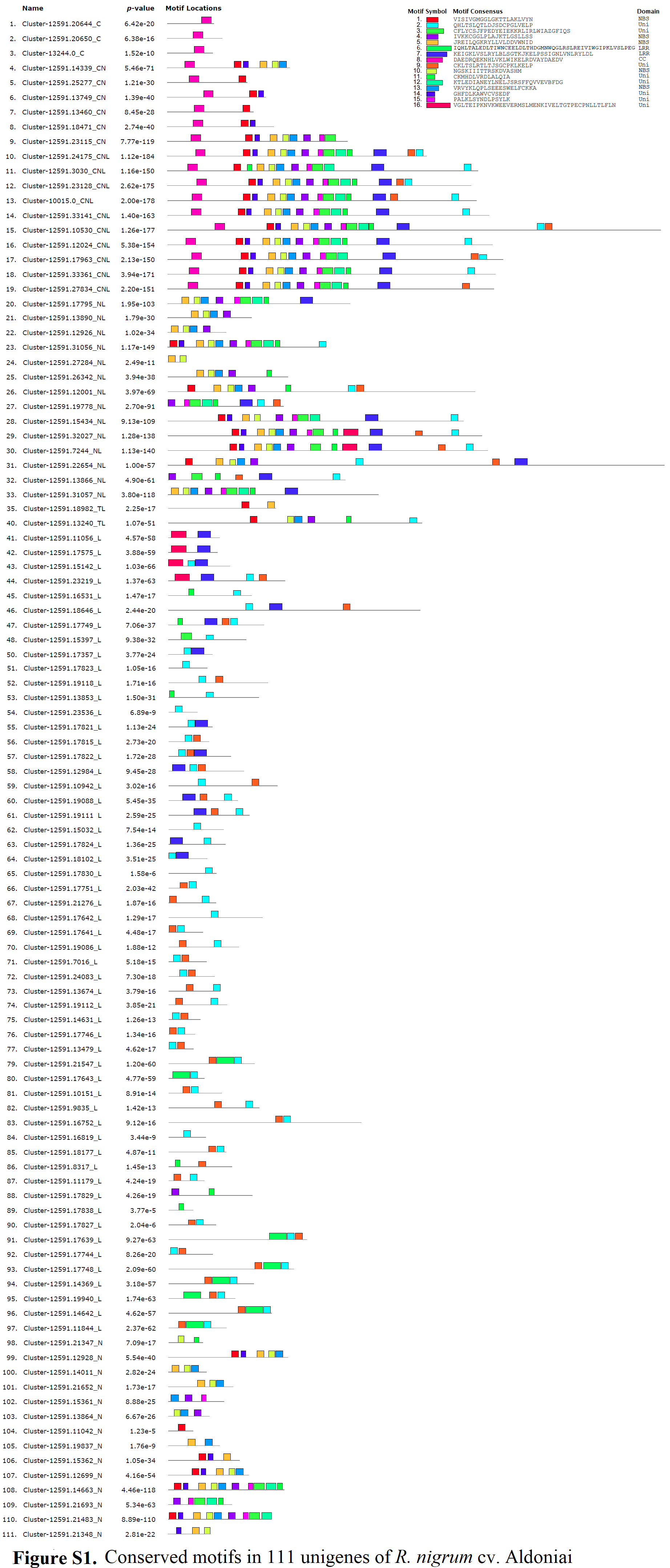

Supplement: Supplementary file 1 [file plants-11-03137-s001.zip › Figure S1.png]
